# Reprogramming anchorage dependency by adherent-to-suspension transition promotes metastatic dissemination

**DOI:** 10.1186/s12943-023-01753-7

**Published:** 2023-03-30

**Authors:** Hyunbin D. Huh, Yujin Sub, Jongwook Oh, Ye Eun Kim, Ju Young Lee, Hwa-Ryeon Kim, Soyeon Lee, Hannah Lee, Sehyung Pak, Sebastian E. Amos, Danielle Vahala, Jae Hyung Park, Ji Eun Shin, So Yeon Park, Han Sang Kim, Young Hoon Roh, Han-Woong Lee, Kun-Liang Guan, Yu Suk Choi, Joon Jeong, Junjeong Choi, Jae-Seok Roe, Heon Yung Gee, Hyun Woo Park

**Affiliations:** 1 Department of Biochemistry, College of Life Science and Biotechnology, Brain Korea 21 Project, Yonsei University, Seoul, 03722 Republic of Korea; 2 Department of Pharmacology, Graduate School of Medical Science, Brain Korea 21 Project, Yonsei University College of Medicine, Seoul, 03722 Republic of Korea; 3 Cytogen, Seoul, Republic of Korea; 4 School of Human Sciences, University of Western Australia, Crawley, WA 6009 Australia; 5 Yonsei Cancer Center, Division of Medical Oncology, Department of Internal Medicine, Brain Korea 21 Plus Project for Medical Sciences, Severance Biomedical Science Institute, Yonsei University College of Medicine, Seoul, 03722 Republic of Korea; 6 Department of Biotechnology, College of Life Science and Biotechnology, Yonsei University, Seoul, 03722 Republic of Korea; 7 Department of Pharmacology and Moores Cancer Center, University of California San Diego, La Jolla, CA 92093 USA; 8 Departments of Surgery, Gangnam Severance Hospital, Yonsei University College of Medicine, Seoul, 06273 Republic of Korea; 9 College of Pharmacy, Yonsei Institute of Pharmaceutical Sciences, Yonsei University, Incheon, Republic of Korea

## Abstract

**Background:**

Although metastasis is the foremost cause of cancer-related death, a specialized mechanism that reprograms anchorage dependency of solid tumor cells into circulating tumor cells (CTCs) during metastatic dissemination remains a critical area of challenge.

**Methods:**

We analyzed blood cell-specific transcripts and selected key Adherent-to-Suspension Transition (AST) factors that are competent to reprogram anchorage dependency of adherent cells into suspension cells in an inducible and reversible manner. The mechanisms of AST were evaluated by a series of in vitro and in vivo assays. Paired samples of primary tumors, CTCs, and metastatic tumors were collected from breast cancer and melanoma mouse xenograft models and patients with de novo metastasis. Analyses of single-cell RNA sequencing (scRNA-seq) and tissue staining were performed to validate the role of AST factors in CTCs. Loss-of-function experiments were performed by shRNA knockdown, gene editing, and pharmacological inhibition to block metastasis and prolong survival.

**Results:**

We discovered a biological phenomenon referred to as AST that reprograms adherent cells into suspension cells via defined hematopoietic transcriptional regulators, which are hijacked by solid tumor cells to disseminate into CTCs. Induction of AST in adherent cells 1) suppress global integrin/ECM gene expression via Hippo-YAP/TEAD inhibition to evoke spontaneous cell–matrix dissociation and 2) upregulate globin genes that prevent oxidative stress to acquire anoikis resistance, in the absence of lineage differentiation. During dissemination, we uncover the critical roles of AST factors in CTCs derived from patients with de novo metastasis and mouse models. Pharmacological blockade of AST factors via thalidomide derivatives in breast cancer and melanoma cells abrogated CTC formation and suppressed lung metastases without affecting the primary tumor growth.

**Conclusion:**

We demonstrate that suspension cells can directly arise from adherent cells by the addition of defined hematopoietic factors that confer metastatic traits. Furthermore, our findings expand the prevailing cancer treatment paradigm toward direct intervention within the metastatic spread of cancer.

**Supplementary Information:**

The online version contains supplementary material available at 10.1186/s12943-023-01753-7.

## Introduction

Although metastasis is the foremost cause of cancer-related death, a specialized mechanism that reprograms anchorage dependency in disseminating tumor cells, the precursors for metastasis, remains a critical area of challenge. Herein, we aim to establish a paradigm referred to as “Adherent-to-Suspension Transition” (AST), which explores undescribed elements and mechanisms involved in reprogramming anchorage dependency of solid tumor cells during the dissemination of circulating tumor cells (CTCs). While extensive effort has been brought into reprogramming cell lineages in the last decade led by the discovery of transcription factors capable of inducing pluripotency and lineage commitments [1, 2], research on reprogramming fundamental cell fates, such as cell morphology and anchorage dependency, remain largely unexplored. To date, research have centered on resolving the mechanisms of epithelial-to-mesenchymal transition (EMT) in various biological context, including embryogenesis, wound healing, and tumor progression [3], that guides the morphological transition between epithelial (E), and mesenchymal (M) phenotypes, both of which are adherent (A) cell types with distinct cell–cell interactions. We asked, however, whether reprogramming the anchorage dependency, or cell–matrix interactions, between adherent (A) and suspension (S) cell types can be directly achieved by the introduction of defined factors, and how that can lead to CTC formation during the dissemination of solid tumor cells.

Herein, we identified a repertoire of transcriptional regulators primarily expressed in the hematopoietic lineages and narrowed down to four key factors, IKZF1, NFE2, BTG2, and IRF8, which we designated AST factors, that are competent to cooperatively reprogram the anchorage dependency of adherent cells into suspension cells. Apart from their known functions in hematopoietic cell types, we show that the induction of AST factors directly converts adherent cells into suspension cells by eliciting spontaneous cell rounding, cell–matrix dissociation, and anoikis resistance without any apparent lineage commitment, altered cell proliferation, and cell–cell interactions. Emerging evidence suggests that cancer cells can adapt specialized properties of different cell types, such as the vascular [4], neuron [5], and immune cells [6] to facilitate tumor progression. It is not known, however, whether cancer cells acquire specialized mechanisms derived from hematopoietic cells to reprogram their anchorage dependency and disseminate into the bloodstream.

CTCs hold the key to understanding critical pathways that mediate the dissemination of cancer [7, 8], however, defined factors that lead to the generation of CTCs from primary tumor are unknown. Here, we show that the induction of AST factors plays critical roles in circulating tumor cells (CTCs) of breast cancer and melanoma cells derived from patients with de novo metastasis and mouse models. Importantly, suppression of metastasis via loss-of-function experiments and pharmacological blockade of AST factors demonstrates their unprecedented metastatic potential during the dissemination processes of solid tumors that can be exploited to develop effective anti-metastatic strategies. Our study establishes a framework how solid tumor cells hijack defined hematopoietic transcriptional regulators to induce adherent-to-suspension transition that leads to the bloodborne dissemination of cancer cells.

## Results

### AST factors reprogram the anchorage dependency of adherent cells

**Fig. 1:**
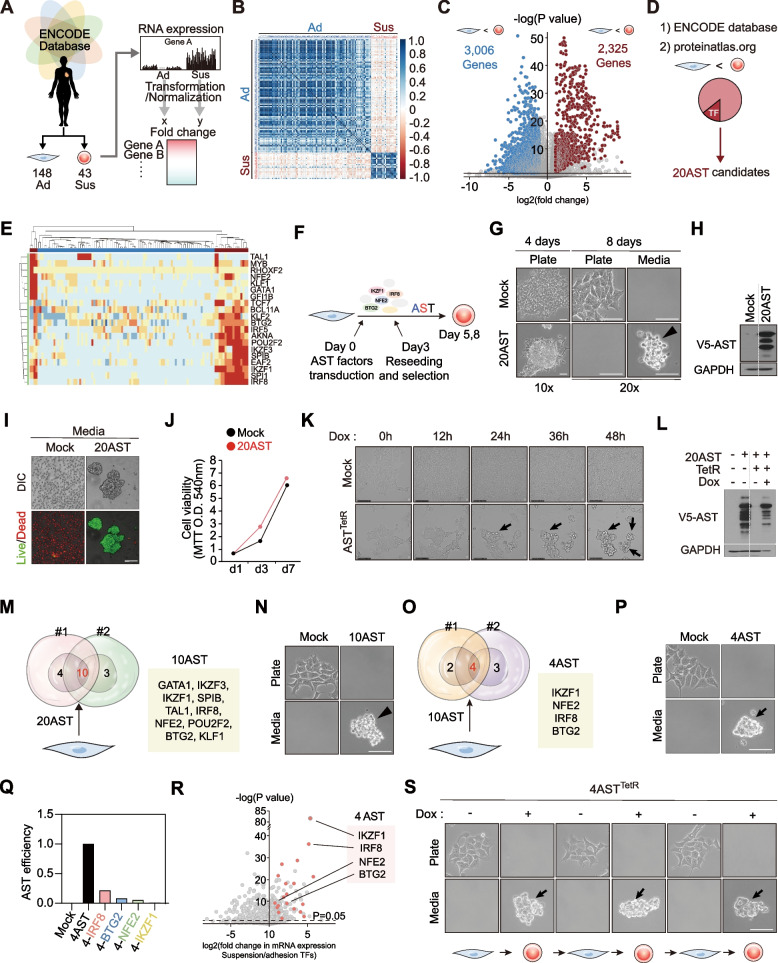
AST factors reprogram anchorage dependency. **A** Schematic of the transcriptomic analysis of 141 adhesion cells and 39 suspension cell lines using RNA expression data from the ENCODE and The Human Protein Atlas databases to identify anchorage-dependent differentially expressed gene profile. **B** Correlation plot of gene expression in adhesion cells versus suspension cells. Correlations were calculated using the Pearson method. **C** Volcano plot showing differentially expressed genes selected based on |fold changes|> 2, |differences|> 1, and *p*-values < 0.05. **D** Selection of 20 hematopoietic transcriptional regulators as candidate AST factors. **E** Heatmap of the 20 candidate AST factors in 90 adhesion and 23 suspension cell lines. **F** Schematic overview of the experimental design for generating HEK293A-20AST cells by lentiviral expression of AST factors. **G**, Representative cell images of HEK293A-mock or 20AST cell morphology in culture plate and media. **H** Immunoblotting analysis of the expression of V5-tagged AST factors in HEK293A-20AST cells. **I** Representative fluorescence images of cell viability in cell culture media of HEK293A-mock and 20AST cells stained using LIVE/DEAD cell imaging assay. Green, Live; Red, Dead. Scale bar, 100 μm. **J** Measurements of cell proliferation rate between adherent mock and suspended 20 AST cells. **K** Images of time-lapse microscopy showing spontaneous cell detachment of HEK293A-4AST^TetR^ cells upon doxycycline treatment. **L** Immunoblotting analysis of the expression of V5-tagged AST factors in HEK293A-20AST^TetR^ cells upon doxycycline treatment **M-P** Schematic for narrowing down from 20 to 10 (M), and then from 10 to 4 (N) AST candidates via multiple trials of AST induction. Representative cell images of HEK293A-10AST (N) or 4AST (P) cells in culture plates and media. **Q** Measurement of AST efficiency by subtracting individual factors from 4 AST factors. **R** Volcano plot showing mRNA expression of transcription factors. x-axis, contrast in expression between suspension and adhesion cells; y-axis, *p*-value. 20 candidate AST factors (orange) were statistically upregulated in suspension cells versus adhesion cells. Upregulation, fold changes > 2, *p*-values < 0.05. **S** Consecutive reprogramming of anchorage dependency in HEK293A-AST^TetR^ cells by the addition and removal of doxycycline. Scale bars, 50 μm

We hypothesized that specific genes differentially expressed between adherent and suspension cell types would be candidates for contributing to the distinct anchorage dependency of each cell type. Hence, we analyzed the gene expression profiles of wide range of cell types that included 141 adherent and 39 suspension cell lines from publicly available databases [9, 10] (Fig. 1A, Tables S1 and S2). The transcriptome analysis showed negative correlation and a discrete pattern between adherent and suspension cell types (Fig. 1B, S1A-C). Notably, over thousands of differentially expressed genes were identified between the two cell types (Fig. 1C, S1D). Gene ontology analyses indicate enrichment of genes related to cell adhesion, cell junctions, and extracellular matrix (ECM) organization in adhesion cell types, whereas suspension cell types showed enrichment of immune related genes (Fig. S1E). In our effort to identify defined factors that could directly convert adherent cells into suspension cells via the induction of anchorage-independent growth, we selected 20 candidates, which we designated as candidate AST factors, by focusing on transcriptional regulators primarily expressed within the hematopoietic lineages (Fig. 1D-E, S1F, Table S3). Since the transcripts of these candidate AST factors are hardly detectable in adherent cells, the biological consequences of their aberrant expression in these cells are unknown. We first asked whether ectopic expression of AST factor candidates into adherent cells can reprogram their anchorage-dependent growth phenotypes. Lentiviral plasmids of 20 AST candidates were used to stably transduce adherent HEK293A cells (Fig. 1F). Remarkably, 20 AST factor expression evoked spontaneous cell rounding followed by complete loss of cell–matrix interactions within 7 days after infection (Fig. 1G-H). Detachment of adherent cells undergo programmed cell death termed anoikis, however, the comparable proliferation rate shown between adherent and AST-induced suspension cells indicates that AST factors confer anoikis resistance to these cells (Fig. 1I-J). These results show that the reprogramming of anchorage dependency of adherent cells into suspension cells is a cell autonomous process that could be achieved by a defined set of hematopoietic transcription factors. We further generated a chemical-inducible HEK293A-20AST^TetR^ cell line, of which we can efficiently control the expression levels of AST factors. Upon doxycycline treatment, induction of AST factors converted adherent HEK293A cells into suspension cells (Fig. 1K-L).

To identify key factors required for reprogramming anchorage dependency, we harvested the suspended cells that have successfully undergone AST and analyzed the expression levels of each factor. By selecting the highly overlapping factors enriched in detached cells from parallel experiments, we were able to narrow the list from 20 factors down to 10 (Fig. 1M-N), and finally to a minimum set of four factors that consist of IKZF1, NFE2, BTG2, and IRF8 (hereinafter referred to as 4AST factors) (Fig. 1O-P, S2A-B). The expression of any single AST factor failed to upregulate the levels of other components and the efficiency of AST induction was dramatically reduced when any of the four factors were excluded, suggesting that the four factors cooperate to achieve AST (Fig. 1Q and S2C). Notably, expressing the omitted sets of AST candidates, of which were excluded from consecutive trials, failed to evoke AST-like morphological changes, suggesting that only specific factors are competent to reprogram anchorage dependency (Fig. S2D-E). Results of chromatin immunoprecipitation and sequencing (ChIP-seq) analysis indicate that, in contrast to suspension cells, adherent cells show depletion of activation marker histone H3 lysine 27 (H3K27) acetylation and enrichment of repressive marker H3K27 methylation in the promoter regions of the 4AST factors, which explains their absence in adherent cells (Fig. S2F-G). Thus, we finalize the combination of transcriptional regulators IKZF1, NFE2, BTG2, and IRF8 as the first essential 4AST factors that endow adherent cells with anchorage-independent growth (Fig. 1R).

Next, we asked whether AST factor-induced spontaneous cell suspension was mediated via reprogramming of adherent cells toward a certain hematopoietic lineage. Despite their roles in hematopoiesis, the expression of 4AST factors did not induce typical blood cell markers or gene signatures, suggesting that 4AST factors do not induce lineage differentiation into blood cells (Fig. S2H). The lack of hematopoietic lineage commitment during AST processes led us to ask whether AST is a readily reversible process. To test the plasticity of AST, we generated HEK293A-4AST^TetR^ cells that were subjected to consecutive cycles of doxycycline treatments (Fig. 1S). We found that HEK293A-4AST^TetR^ cells treated with doxycycline exhibited cellular detachment that was reversible upon doxycycline withdrawal, and that this cycle could be repeated multiple times (Fig. 1S). These results demonstrate that AST factors can reprogram the anchorage dependency of adherent cells in a reversible manner and the dynamic plasticity of AST depends simply on their expression levels.

### AST factor-mediated YAP-TEAD suppression dissociates cell–matrix interaction

**Fig. 2:**
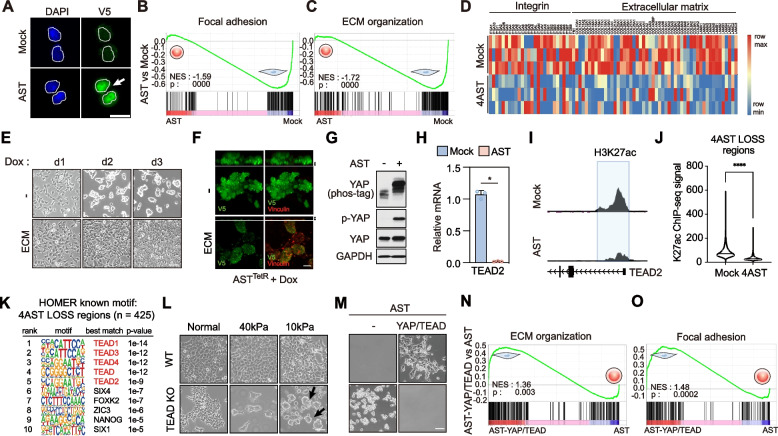
AST factor-mediated YAP-TEAD suppression dissociates cell–matrix interaction. **A** Immunofluorescence analysis of nuclear expression of ectopic V5-tagged 4 AST factors in HEK293A-4AST cells. Scale bar, 20 μm. **B-D** GSEA and heatmap of focal adhesion- and ECM-related genes in HEK293A-4AST cells compared to control cells. **E–F** Effect of restoring ECM (E, fibronectin) and focal adhesions (F, green, V5; red, vinculin) in doxycycline-induced HEK293A-AST^TetR^ cells. Scale bar 50 μm (E), 10 μm (F), Upper images; z-section, scale bar, 5 μm (F). **G** Immunoblotting analysis of AST factor-induced phosphorylation and inhibition of YAP. **H** Quantitative real-time PCR analysis of the TEAD2 transcript levels in 4 AST cells. **I** Analysis of H3K27ac ChIP-seq profiles at TEAD2 locus in HEK293A-mock and AST cells. **J** Analysis of H3K27ac ChIP-seq signal at the 4AST LOSS region in AST cells. Mean values are shown with error bars representing the s.d. of *n* = 3 independent replicates. *p < 0.05. **K** List of the top ten known motifs enriched at AST LOSS regions (*n* = 425). **L** Representative images of partial AST induction in TEAD KO cells cultured in low stiffness plates (40 kPa or 10 kPa). Scale bar, 50 μm. **M** Restoration of anchorage dependency in AST cells by ectopic expression of TEAD2 and constitutively active YAP-S127A. Scale bar, 50 μm. **N–O** Restoration of focal adhesion- and ECM-related gene signatures in HEK293A-4AST cells by reconstitution of TEAD2 and constitutively active YAP-S127A

Phenotypically, we categorized the mechanism of AST into largely a two-step process. AST confers anchorage-independent growth by eliciting 1) spontaneous cell–matrix dissociation and 2) acquisition of anoikis resistance. Because AST factors are transcriptional regulators localized in the nucleus, we investigated specific transcriptional activities that underlie AST (Fig. 2A). Consistent with suspension cell profiles, one of the most prominent gene signatures in HEK293A-4AST cells was the global suppression of genes involved in focal adhesion assembly and integrin-ECM interactions (Fig. 2B-D, S3A-B). In adherent cells, integrin-ECM interactions at focal adhesions mediate cell–matrix adhesion. Hence, the global suppression of integrin αβ subunits as well as other major ECM proteins provide mechanistic insights into AST-induced spontaneous cell detachment. Phenotypically, we found that HEK293A-4AST cells continued to grow in clusters, suggesting that AST factors suppress cell–matrix interactions, but not cell–cell interactions. To further confirm whether loss of integrin-ECM interaction is a major determinant of AST factor-mediated cell detachment, we reinforced focal adhesion assemblies by culturing cells in ECM-coated plates such as fibronectin and then induced AST (Fig. 2E). The enhancement of cell-ECM interactions dramatically hindered doxycycline-induced cell–matrix dissociation by upregulating focal adhesion assemblies shown by vinculin staining in HEK293A-4AST^TetR^ cells, suggesting that the loss of ECM-integrin adhesion is a critical process of AST (Fig. 2F).

The transcriptional coactivator YAP is a well-established oncogenic effector of the Hippo pathway that binds transcription factor TEAD to promote cell-ECM interactions in cancer cells by upregulating integrin and ECM genes [11–14]. As such, YAP-TEAD axis exhibits tumor suppressive activity in certain contexts. Similar to IKZF1-mediated repression of YAP-TEAD during pre-B cell differentiation [15], we found that AST factors dramatically suppressed the expression of YAP-TEAD target genes and Hippo-related gene signatures by inducing inhibitory YAP^S127^ phosphorylation by LATS kinases (Fig. 2G, S3A-C) and reducing the expression and H3K27ac at the TEAD2 locus (Fig. 2H-I). We tested whether YAP inactivation and loss of TEAD expression results in impaired target gene expression. ChIP analysis indicates that chromatin regions where H3K27ac accumulation was reduced showed marked enrichment of canonical TEAD-binding DNA motifs (Fig. 2J-K). Consistent with these results, TEAD depletion alone was sufficient to elicit partial AST phenotypes shown by spontaneous cell rounding and detachment when TEAD KO cells were cultured on low stiffness plates (Fig. 2L). Next, we restored YAP-TEAD deficiency to demonstrate whether AST factor-mediated YAP-TEAD suppression is a prerequisite for AST. Remarkably, we observed a recovery of cell attachment through restoration of focal adhesion assembly and integrin-ECM interactions when AST cells were reconstituted with TEAD and constitutively active YAP^S127A^ (Fig. 2M-O). Thus, the antagonism between AST factors and adhesive transcriptional regulators, such as YAP-TEAD, comprise a key mechanism that governs the reprogramming of anchorage dependency.

Because AST is achieved via loss of cell–matrix adhesion, it is conceivable to speculate that AST could arise in both adherent cell types regardless of their epithelial or mesenchymal characteristics. To test whether AST reprograms anchorage dependency independent of EMT and cell–cell contacts, we investigated SUIT2 cells that harbor epithelial phenotypes and express markers such as E-cadherin. We found that ectopic expression of 4AST factors induced spontaneous cell–matrix detachment without altering the expression of typical EMT markers, such as E-cadherin, N-cadherin, or vimentin (Fig. S3D-E). Moreover, E-cadherin remained localized on the cell surface between cell–cell junctions in AST-induced suspension cell clusters (Fig. S3F). These results demonstrate that AST can occur directly from either epithelial or mesenchymal cell types without altering their cell–cell contacts.

### AST factor-mediated globin induction confers anoikis resistance

**Fig. 3:**
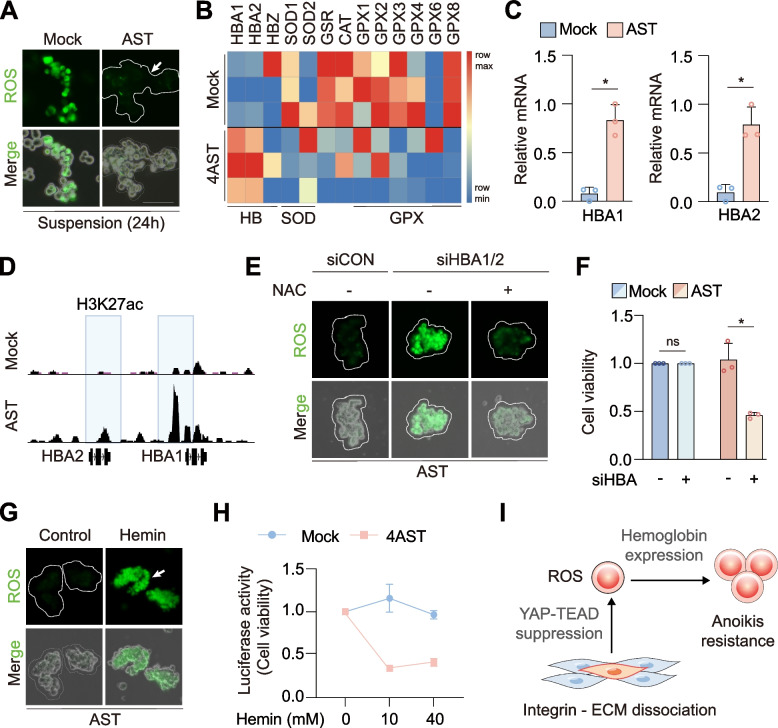
AST factor-mediated globin induction confers anoikis resistance. **A** Measurement of cell detachment-induced ROS production in HEK293A-4AST (right) versus trypsinized cells (left). Green, ROS. Scale bar, 50 μm. **B** Heatmap of genes involved in ROS defense mechanisms in HEK293A-mock and 4AST cells. **C** Quantitative real-time PCR analysis of the HBA1/2 transcript levels in 4AST cells. **D** Analysis of H3K27ac ChIP-seq profiles at HBA1 and HBA2 loci in HEK293A-mock and 4AST cells. **E** Representative fluorescence images of ROS accumulation in HEK293A-4AST cells treated with scramble or HBA1/2 siRNAs with or without N-acetylcysteine (NAC). **F** Measurement of cell viability of HEK293A-4AST cells upon siRNA-mediated HBA1/2 depletion. **G** Fluorescence images of ROS accumulation in HEK293A-4AST cells by hemin treatment. Scale bar, 50 μm. **H** Measurement of cell viability in HEK293A-4AST cells upon hemin treatment. *n* = 3. **I** Schematic of mechanistic insights into AST factor-mediated reprogramming of anchorage dependency. For (C and F) mean values are shown with error bars representing the s.d. of *n* = 3 independent replicates. *p < 0.05

Anoikis resistance upon cell detachment is required for the proliferation of suspended cells that had undergone AST. Typically, the loss of cell–matrix adhesion induces anoikis via oxidative stress that is associated with increased intracellular reactive oxygen species (ROS) [16]. To determine whether AST factors are sufficient to relieve oxidative stress triggered by cellular detachment, we measured intracellular ROS levels in suspended cells. In contrast to trypsin-induced cell detachment, which showed strong induction of ROS, AST factor-mediated cell detachment was associated with significantly lower level of ROS (Fig. 3A). We searched for genes that could protect AST cells from ROS-induced anoikis, including family members of the hemoglobin (HB), superoxide dismutase (SOD), and glutathione peroxidase (GPX), and found that AST-induced suspension cells specifically expressed significantly higher levels of HBA1 and HBA2, which encode α-globin chains expressed primarily in red blood cells (Fig. 3B-C). Increased HBA1/2 expression was associated with the gain of H3K27 acetylation at each locus (Fig. 3D). Notably, circulating tumor cells (CTCs) that are disseminated from primary tumors have been shown to express certain isoforms of hemoglobin genes to suppress intracellular ROS and cell death [17, 18]. To investigate the role of HBA1/2 in AST-induced anoikis resistance, we depleted HBA1/2 via siRNA knockdown (Fig. 3E-F) or hemin treatment (Fig. 3G-H). We found that downregulation of HBA1/2 induced anoikis in HEK293A-4AST cells, but not in control adherent cells, by marked accumulation of ROS that was suppressed by antioxidant treatment. These results suggest that AST factor-mediated HBA1/2 induction contributes to anoikis resistance in suspension cells. Together, we elucidate the molecular mechanisms underlying AST factor-mediated reprogramming of anchorage dependency via 1) the suppression of YAP-TEAD axis to dissociate cell–matrix adhesion, and 2) the induction of HBA1/2 to acquire resistance toward ROS-induced anoikis (Fig. 3I).

### Induction of AST factors in the metastatic dissemination of CTCs

**Fig. 4:**
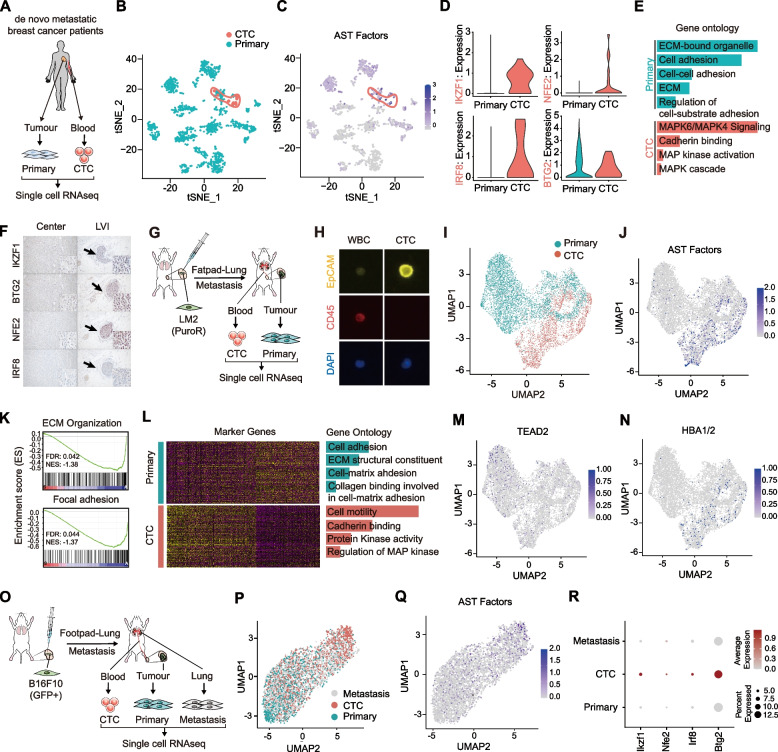
Induction of AST factors in disseminated breast cancer CTCs. **A** Schematic overview of the isolation and scRNA-seq analysis of primary tumor cells and CTCs from de novo metastatic breast cancer patients. CTCs were isolated using HDM chips. **B** tSNE plot representing 1,509 cancer cells (EpCAM^+^) sorted from breast cancer tissues and PBMC of three de novo metastatic breast cancer patients analyzed by scRNA-seq. Turquoise dots, 1,488 cells from breast tissues; orange dots, 21 CTCs from blood samples. CTCs are in region marked with orange boundary. **C**, Scaled and aggregated transcriptional expression of 4AST factor genes. Cell positions are based on tSNE plot in (B). **D** Violin plots showing normalized expression levels of IKZF1, IRF8, NFE2, and BTG2 in each group of (B). **E** Bar chart showing gene ontology (GO) analysis of differentially expressed genes (DEGs) between primary tumor cells and CTCs. Length of each bar, FDR-corrected *p*-value of each term. **F** Representative histologic images of AST factor expression in lymphovascular invasion (LVI) from primary human breast cancer patients. **G** Schematic overview of the isolation and scRNA-seq analysis of LM2-derived primary tumor cells and CTCs from orthotopic model of breast cancer metastasis. PuroR^+^-LM2 cells were injected into mammary fat pads of NSG mice and CTCs were subsequently isolated using HDM chips. **H** Immunofluorescence analysis of EpCAM^+^ and CD45^−^ CTCs. Yellow, EpCAM; red, CD45. **I** UMAP embedding of analyzed transcriptomes of 3,006 CTCs (orange) and 5,511 primary tumor cells (turquoise). **J** Scaled and aggregated transcriptional expression of 4AST factor genes. Cell positions are from the UMAP plot in (I). **K** GSEA of ECM organization- and focal adhesion-related genes in CTCs compared to primary tumor cells. **L** Heatmap of GO analysis of 105 and 100 genes differentially expressed in CTCs and primary tumor cells. **M–N** Feature plots of cells showing the expression levels of TEAD2 (M) and HBA1/2 (N). Cell positions are from the UMAP plot in (l). **O** Schematic showing the procedure used for the isolation and scRNA-seq analysis of B16F10-derived primary tumor cells, CTCs, and metastatic tumor cells from an orthotopic model of melanoma metastasis. GFP-positive cells were injected into the footpads of C57BL/6N mice and tumor cells were subsequently isolated using FACS. **P** UMAP embedding of analyzed transcriptomes of 1,139 CTCs (orange), 1,129 primary tumor cells (turquoise) and 1,630 metastatic tumor cells (light grey). **Q** Feature plots of cells showing the expression levels of 4AST factor genes. **R** Dot plot showing the average expression level of the 4 factors, Btg2, Ikzf1, Irf8, and Nfe2, in primary tumor, CTCs, and metastasized lung nodules, respectively

Although thus far we have explored the consequences of ectopic expression of AST factors in adherent cell lines, we hypothesize that AST is a physiologically relevant process that function in the dynamic modulation of cell morphology and anchorage dependency in vivo. Hence, we investigated the in vivo relevance of AST paradigm in the process of metastatic dissemination and survival of circulating tumor cells (CTCs) derived from solid tumors such as breast cancer and melanoma cells. First, to investigate the human relevance of AST, we enrolled de novo metastatic breast cancer patients and obtained paired samples from each patient that consist of breast tissues and blood cells (Fig. 4A). Importantly, it is highly conceivable that captured CTCs derived from untreated patients with de novo metastatic lesions would harbor strong metastatic potentials. To investigate the expression level of AST factors in human CTCs, cells were collected from whole blood using size exclusion based HDM (high density microporous) chips and further processed via single-cell RNA sequencing (scRNA-seq) (Fig. 4B). After filtering the cells with quality control measures and correcting for batch effects, we isolated 55,580 and 15,142 single cells from blood and breast cancer tissues, respectively, from three patients and visualized the results on t-stochastic neighbor embedding (t-SNE) plots (Fig. S4A-B). Cell type annotation indicate that breast cancer tissues consist of epithelial, endothelial, and lymphoid cell types (Fig. S4C-D), whereas blood samples consist of lymphocytes with minor population of epithelial cells that largely showed mutually exclusive expression patterns between EpCAM and PTPRC (CD45) markers (Fig. S4E-F). We identified a total of 21 EpCAM^+^ CTCs collected from 3 patients. Remarkably, expression levels of key AST factors, IKZF1, NFE2, and IRF8, were significantly enriched in CTCs that were, however, largely absent in primary tumor cells (Fig. 4C-D). Consistent with in vitro results, we observed downregulation of cell-substrate adhesion and ECM components in CTCs compared to primary tumor cells (Fig. 4E). Next, we validated the expression level of endogenous AST factors in human breast cancer patients with frequent lymphovascular invasion (LVI) that represent tissue-equivalent lesions of CTCs. Consistently, AST factors showed much stronger nuclear expression in these patient-derived lymphatic tumor cells than in the primary tumors (Fig. 4F, S5A). Notably, aberrant expression of several key AST factors in CTCs was also evident in previous studies of human breast cancer patients as well as pancreatic cancer and melanoma mouse models [19–21] (Fig. S5B-D).

Next, to overcome the small number of isolated CTCs and confirm the results obtained from human patients, we performed mouse orthotopic fat pad injection with LM2 breast cancer cells, a derivative of MDA-MB-231 cells selected for their ability to metastasize to the lung [22]. CTCs were collected from whole blood using HDM chips and processed via scRNA-seq (Fig. 4G-H). To maximize the purity of CTC population, we introduced PLKO vector into LM2 cells, then analyzed scRNA-seq data of CTCs that were positive for the PLKO vector-derived puromycin-resistant (puroR^+^) gene transcript. After filtering the cells with quality control measures and correcting for batch effects, we visualized the sequencing data of 5,511 cells from primary tumors and 3,006 CTCs using the Uniform Manifold Approximation and Projection (UMAP) (Fig. 4I). PuroR^+^ CTCs consist of both EpCAM^+^ and EpCAM^−^ CTC populations [23] (Fig. S6A). In this analysis, we annotated 6 cell types and classified these into 14 distinct clusters (Fig. S6B-D). We found clusters 4, 5, and 7, which are enriched with CTCs, mostly consist of cell types annotated as epithelial cells or mesenchymal stem cells, suggesting that, similar to previous reports, both cell types give rise to most of the CTCs derived from breast cancer cells [24, 25] (Fig. S6E).

Because CTCs are rare subpopulation of tumor cells competent to disseminate and survive in the blood stream, we expected that CTCs harbor transcriptional profiles distinct from their primary counterpart. Despite partial overlap, we observed clustering of CTCs apart from primary tumor cells (Fig. 4I). This indicates that the process of cellular dissemination coincides at least in part with transcriptional reprogramming, however, the extent to which intrinsic or environmental factors contribute to such transcriptomic heterogeneity warrants further investigation. Next, we asked whether the molecular mechanisms of AST uncovered in vitro are recapitulated during breast cancer cell dissemination in vivo. A significant population of CTCs showed markedly increased levels of any one of the endogenous AST factors including IKZF1, NFE2, and BTG2. In contrast, AST factors were largely absent from the primary tumor cells (Fig. 4J and S6F). Consistent with the results from breast cancer patient-derived CTCs, we observed downregulation of focal adhesion, cell–matrix adhesion, and ECM components (Fig. 4K-L) as well as suppression of TEAD2 in CTC clusters relative to their primary tumor counterparts (Fig. 4M). Moreover, CTCs exhibited higher expression of HBA1/2, which is consistent with our finding that ectopic expression of the AST factors induced HBA1/2 in AST cells and with recent reports showing that hemoglobin facilitates breast cancer CTCs to suppress anoikis and form distant metastases [17, 18] (Fig. 4N, S5C).

Next, we recapitulated these results in melanoma, the most invasive skin cancer with the highest risk of death. We performed orthotopic footpad injections with B16F10 melanoma cells in C57BL/6N mice, and then we isolated primary tumor cells, CTCs, and metastatic tumor cells from lung nodules for scRNA-seq analyses (Fig. 4O). After FACS sorting GFP-positive B16F10 cells, we visualized the transcriptome of 1,129 primary tumor cells, 1,139 CTCs, and 1,630 metastatic tumor cells (Fig. 4P) that mainly consisted of melanocyte markers (Fig. S6G-H). Remarkably, we found dynamic expression of AST factors from dissemination to colonization in the metastatic cascade. In line with breast cancer CTCs, the melanoma CTCs showed upregulation of AST factors, which were absent in the primary tumor cells. Furthermore, we found that colonized lung nodules showed reduced expression of AST factors to levels comparable to that of the primary tumor cells (Fig. 4Q-R), indicating that the plasticity of AST in cancer cells correlates with their anchorage dependency.

Collectively, the induction of key AST factors and the reprogramming mechanisms of anchorage dependency were recapitulated in disseminated breast cancer and melanoma CTCs. These results demonstrate in vivo that CTCs could hijack specific hematopoietic programs through the spatial and temporal modulation of AST factors to disseminate from primary sites and survive within the circulation.

### Targeting AST factors suppress cancer cell dissemination and metastasis

**Fig. 5:**
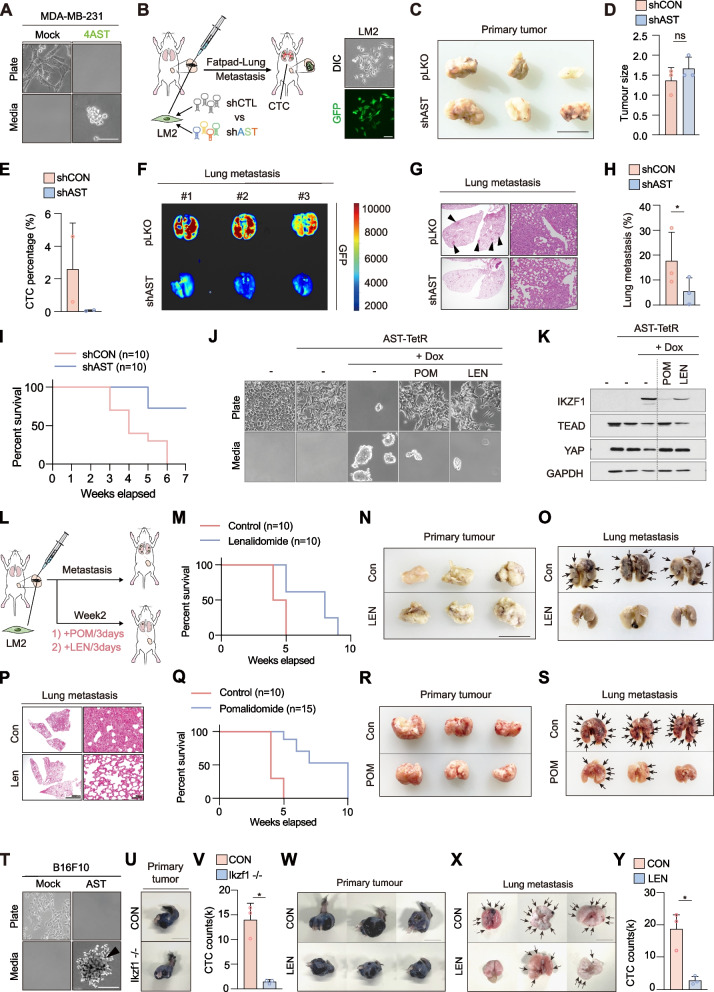
Targeting AST factors suppress cancer cell dissemination and metastasis. **A** Representative images of MDA-MB-231-mock versus 4AST cell morphology. Scale bar, 50 μm. **B** Schematic overview of mammary fat pad xenografts of 4AST factor depleted LM2 cells. **C** Representative images of primary tumors dissected after injection of mock or 4AST factor depleted LM2 cells. **D** Measurement of primary tumor growth in LM2-mock versus 4AST factor-depleted tumors. *n* = 3; ns, not significant. **E** Measurement of EpCAM^+^/CD45^−^ CTC counts normalized by total blood cells from duplicated slides harvested from control and 4AST factor-depleted LM2 baring mice. **F** Bioluminescence ex vivo images of lung metastases after LM2 fat pad injection. **G-H** Measurement of lung metastases quantified by the number of metastatic lung nodules derived from mock versus AST factor-depleted LM2 cells. **I** Kaplan–Meier survival plot showing the survival rates of mice injected with mock or 4AST factor-depleted LM2 cells, *n* = 10. **J** Images of HEK293A-AST^TetR^ cells co-treated with pomalidomide (100 μM) or lenalidomide (50 μM) after doxycycline-mediated AST induction for 3 days. Scale bar, 50 μm. **K** Blockade of IKZF1 accumulation in doxycycline-induced HEK293A-AST^TetR^ cells co-treated with pomalidomide or lenalidomide. **L** Schematic of the orthotopic breast tumor model subjected to intraperitoneal injection of pomalidomide (10 mg/kg) or lenalidomide (10 mg/kg) once every 3 days. **M** Kaplan–Meier survival plot showing the survival rates of LM2-injected mice treated with lenalidomide (10 mg/kg). Control, *n* = 10; lenalidomide; *n* = 10. **N–O** Images of primary tumors (N) and lungs (O) dissected from mice treated with either vehicle or lenalidomide. **P** Measurement of lung metastases by vehicle versus lenalidomide administration quantified by number of metastatic lung nodules. **Q** Kaplan–Meier survival plot showing the survival rates of LM2-injected mice treated with pomalidomide (10 mg/kg). Control, *n* = 10; pomalidomide; *n* = 15. **R-S** Images of primary tumors (R) and lungs (S) dissected from mice treated with either vehicle or pomalidomide. **T** Representative images of B16F10-mock versus 4AST cell morphology. Scale bar, 50 μm. **U** Representative images of primary tumors resulting from B16F10 cell injection into mouse footpad. Above, B16F10-control primary tumor; Below, B16F10-Ikzf1^−/−^-derived primary tumor; Scale bar, 0.5 cm. **V** A bar plot showing the numbers of circulating tumor cells (CTCs) in B16F10-control and -IKZF1^−/−^-injected mouse blood, respectively. Error bars are means ± SD of *n* = 3 independent replicates. *p < 0.05. **W** A bar plot showing the numbers of CTCs in vehicle and lenalidomide-treated mouse blood, respectively. Error bars are means ± SD of *n* = 3 independent replicates. *p < 0.05. **X–Y** Images of primary tumors (X) and lungs (Y) dissected from mice treated with either vehicle or lenalidomide

To determine whether induction of AST factors in CTCs are necessary for the metastatic dissemination of breast cancer cells, we first confirmed that ectopic expression 4AST factors in LM2 cells is sufficient to promote AST in vitro cell culture systems and in vivo CTC formation by orthotopic injection into the mammary fat pad of NOD/SCID gamma (NSG) mice (Fig. 5A and S7A-B). To test whether preventing the induction of AST factors suppress metastatic dissemination, likely through impaired CTC generation, stable knockdown of AST factors was performed in LM2 cells. Since AST factors are absent in adherent breast cancer cells, the knockdown efficiency of each shRNA was validated in K562 blood cancer cell line that endogenously express 4AST factors (Fig. S7C). We orthotopically injected AST factor-depleted and control LM2 cells into the mammary fat pad and measured the primary tumor growth, CTC number, and metastatic burden (Fig. 5B). Notably, because 4AST factors are hardly expressed in adherent cells and primary tumors, depletion of these factors had negligible effects on adherent cell proliferation in culture plates (Fig. S7D-E) as well as LM2-derived primary tumor growth (Fig. 5C-D). Interestingly, although mice from both groups harbored tumors of comparable size, blocking the induction of AST factors significantly reduced the numbers of CTCs in circulation (Fig. 5E), suggesting that AST factor blockade specifically interferes with cancer cell dissemination. Next, we measured the metastatic burden 4 weeks after primary tumor development by quantifying GFP intensity of LM2 cells and by comparing colonized lung nodules from each group (Fig. S7F). We found that blocking the induction of AST factors dramatically reduced the number and size of distant lung metastases (Fig. 5F-H), thus improving long-term survival in mice (Fig. 5I). These results demonstrate that in vivo suppression of AST factors effectively prevents blood-borne metastasis and improves survival rates.

Next, we exploit HEK293A-4AST^TetR^ cells as a platform to verify compounds that target AST factors, thereby interfere with spontaneous dissemination and anoikis resistance. IKZF1, a driver gene in multiple myeloma and other B-cell lymphomas, is the molecular target of IMiDs (immunomodulatory drugs) including lenalidomide and pomalidomide, which facilitate CRBN-mediated degradation of the Ikaros family proteins [26, 27]. Thus, our discovery of 4AST factors as mediators of metastatic dissemination provides molecular rationale to reposition IMiDs as anti-metastatic agents for solid tumors. Remarkably, in HEK293A-4AST^TetR^ cells, doxycycline treatment triggered AST, whereas cells cotreated with lenalidomide or pomalidomide significantly reduced IKZF1 expression and cell dissemination while leaving the remaining adherent population intact and viable (Fig. 5J-K).

To evaluate the anti-metastatic efficacy in vivo, we treated mice with each drug after orthotopic injection of LM2 breast cancer cells (Fig. 5L). Surprisingly, treatment with either lenalidomide (Fig. 5M-P, S7G) or pomalidomide (Fig. 5Q-S) not only reduced lung metastases, but also prolonged survival in mice even with primary tumors of comparable size. Next, we examined the role of AST factors in the development of melanoma CTCs. In B16F10 melanoma cells, ectopic expression of AST factors was sufficient to elicit spontaneous cell suspension (Fig. 5T). Consistent with our findings with breast cancer cells, depletion of IKZF1 (Fig. 5U-V, S7H-J) or lenalidomide treatment (Fig. 5W-Y) greatly reduced the number of CTCs produced from footpad-injected B16F10 cells without affecting the growth of the primary melanoma. These data demonstrate the physiological relevance of AST factors in solid tumor dissemination and provide a rationale to repurpose lenalidomide and pomalidomide as effective anti-metastatic agents by impeding AST factor-induced cancer cell dissemination and reinforcing anchorage dependency.

## Discussion

Depending on cell morphology and anchorage dependency, the many cell types in the human body can be categorize into either adherent cell or suspension cell types. To date, a concept that embraces the reprogramming of adherent cells into suspension cells is yet to be revealed. Here, with the goal to advance our understanding of how to reprogram distinct anchorage dependencies, we uncovered an overarching principle in cell biology and metastatic diseases referred to as AST. We discovered a combination of AST factors, IKZF1, NFE2, BTG2, and IRF8, that are transcriptional regulators primarily expressed in the hematopoietic lineages, which can impair cell adhesive properties and spontaneously dissociate cells from their matrix. In the present study, we have demonstrated the role of AST factors in reprogramming anchorage dependency of certain cell types, however, further expanding the repertoire of AST factors and related mechanisms toward various cell types and diseases will undoubtedly bring new opportunities for future basic research and therapeutic interventions in various human pathophysiological contexts.

Recent studies report that primary and metastatic tumors within individuals harbor common genetic mutations, suggesting that metastasis may rely more on non-genetic tumor-promoting processes such as cellular plasticity or microenvironmental cues than previously recognized [28, 29]. Thus, it is intriguing that the plasticity of fundamental cellular phenotypes, such as drastic changes in cell morphology and anchorage dependency, simply depends on the expression level of AST factors rather than their genetic mutations. To date, no driver mutations within AST factors have been reported in solid tumors. Epigenetic activation of AST factors in adherent cells trigger several unique transcriptional programs, such as global suppression of integrin and ECM components via inhibition of YAP-TEAD axis and induction of anoikis resistance via hemoglobin genes to evoke cell state transition, however, in the absence of lineage differentiation. Thus, AST presents another layer of complexity within the metastatic cascade via context specific induction of the hematopoietic elements in detaching solid tumor cells. The induction of AST factors during the dissemination of tumor cells, but their absence in primary or metastatic tumors, suggests that spatial and temporal microenvironmental stressors, ligands, or tumor-stroma interactions within the invasive front and vasculature could trigger hematopoietic mimicry in solid tumor cells. Furthermore, reprogramming anchorage dependency via AST is a reversible process, therefore, the functional consequence of AST plasticity in the final steps of the metastatic cascade, such as extravasation and colonization, would be an exciting avenue for further investigation.

Although most cancer-related deaths are a result of metastasis, much less information on the pathophysiological mechanisms of metastasis is available than is for the primary tumor. Thus, factors that mediate the blood borne dissemination of cancer, which lead to the active intravasation and CTC generation, are largely unknown. These factors may not be readily evident through analyses of bulk primary or metastatic tumor cells. Employing multi-omics approaches, therefore, in paired patient samples that consist of not only primary tumors and metastatic lesions, but also CTCs isolated from blood samples, would be necessary to explore undescribed mechanisms and therapeutic targets specialized in the dissemination process such as AST factors. In the present study, analyzing such paired samples from de novo metastatic breast cancer patients and mouse models made it possible for us to elucidate the unprecedented AST framework in CTC formation, which were largely invisible during primary tumor development and growth.

In our effort to establish AST factors as effective anti-metastatic targets, we provide the first reference that thalidomide-derivatives, which are used to treat hematologic malignancies such as multiple myeloma [30], targets IKZF1 for degradation [26, 27], thus effectively impair AST factor-induced reprogramming of anchorage dependency in solid tumor cells. These results provide in vivo proof-of-concept that suppressing the induction of AST factors and their target genes in disseminating tumor cells and CTCs can prevent or delay the metastatic outgrowth of cancers. Hence, therapeutic targeting of AST factors opens promising avenues to selectively inhibit metastatic processes that do not rely on primary tumor responses.

Recent evidence indicate that CTC clusters have greater metastatic potential than single CTCs [19, 31, 32]. Multiple cell adhesion molecules and tight junction proteins that confer epithelial-like traits, such as plakoglobin, ICAM1, and CD44, have been identified to underlie the outperforming attributes of CTC clusters in cell survival, cancer stemness, and immune invasion [19, 31, 33, 34]. Moreover, epithelial genes such as E-cadherin and cytokeratin in breast cancer cells has been shown to play critical roles within the metastatic cascade and collective invasion [35, 36]. CTCs can also dynamically change their epithelial or mesenchymal cell types during their response to anticancer therapy and disease progression [24, 25]. Unlike EMT, AST factors selectively dissociate cell–matrix interactions without affecting cell–cell interactions, therefore, AST may underlie the mechanism that confer CTC clusters the ability to detach from the primary tumor while maintaining intact intercellular adhesions as they survive in the blood stream. Furthermore, we show that reprogramming anchorage dependency by AST factors can proceed in regardless of either epithelial or mesenchymal cell types, and consistently, both cell types within the LM2- and B16F10-derived CTC population were found to express AST factors. Therefore, AST paradigm provides an alternative mechanism to perceive EMT-related and -unrelated metastases [35, 37–39].

Expanding the applicability of AST beyond pathophysiological contexts, whether AST could offer advanced technology in the engineering of anchorage independent cell state that enhances the expansion and production rate of protein therapeutics in suspension is under investigation. Moreover, as we continue to explore the antagonistic relationship between YAP-TEAD against AST factor-induced dissemination, it may uncover in part the conundrum of context-dependent oncogenic or tumor-suppressive binary functions of critical cancer driver genes [11]. Clinically, moving beyond the primary tumor-centric point-of-view, our data expand the prevailing cancer treatment paradigm toward direct intervention within the metastatic processes.

## Conclusion

Our findings established an unprecedented framework of hematopoietic mimicry during the metastatic dissemination of CTCs. Here we show that CTCs from mouse models and patients with de novo metastasis hijack defined hematopoietic transcriptional regulators that reprogram anchorage dependency via Adherent-to-Suspension Transition mechanisms. Targeting AST factors specifically blocks CTC generation and metastasis without affecting the primary tumor growth. Therefore, AST provides a novel theoretical basis in cancer metastasis and uncover AST factors as promising therapeutic targets to tackle the dissemination of solid tumor cells.

### Supplementary Information

**Additional file 1: Movie S1.** Induction and reversibility of AST in HEK293A-ASTTetR cells. **Video 1.** Video of HEK293A-ASTTetR cell proliferation in normal culture condition. **Video 2.** Video of HEK293A-ASTTetR cell proliferation after doxycycline treatment. **Video 3.** Video of HEK293A-ASTTetR cell proliferation after doxycycline removal.

**Additional file 2: Figure S1.** Analysis of transcriptional expression patterns in adhesion or suspension cells.

**Additional file 3: Figure S2.** AST factors evoke cell detachment in the absence of lineage differentiation.

**Additional file 4: Figure S3.** Gene signatures and mechanisms of the adherent-to-suspension transition.

**Additional file 5: Figure S4.** scRNA-seq analysis of primary tumors and blood cells from de novo metastatic breast cancer patients.

**Additional file 6: Figure S5.** Induction of AST factors in human and mouse model-derived CTCs and LVI.

**Additional file 7: Figure S6.** scRNA-seq analysis of primary tumor and CTCs from breast cancer and melanoma xenograft mouse model.

**Additional file 8: Figure S7.** Effect of AST factor inhibition in cancer cell proliferation and dissemination.

**Additional file 9: Table S1.** RNA sequencing data of adhesion and suspension cells used in this study from Gene Expression Omnibus (GEO).

**Additional file 10: Table S2.** Microarray data of adhesion and suspension cells used in this study from Human Protein Atlas (HPA).

**Additional file 11: Table S3.** 100 transcription factors upregulated in suspension cells compared to adhesion cells.

## Data Availability

ENCODE RNA-seq data for the adhesion and suspension cell lines were obtained from the ENCODE portal (https://www.encodeproject.org/). The relevant dataset IDs are provided in Table S1. HPA RNA-seq data were obtained from the HPA website (https://www.proteinatlas.org/, RNA HPA cell line gene dataset 22) (Table S2). All data are deposited to Korean Nucleotide Archive (KoNA, https://www.kobic.re.kr/kona/). The bulk RNA-seq data is deposited with the accession number KRA2200722. The single-cell RNA-seq data based on 10X Genomics of breast cancer patients, mouse breast cancer, and melanoma are deposited with accession number KRA2301014, KRA2200726, and KRA2301012, respectively.

## Abbreviations

AST: Adherent-to-Suspension Transition
CTCs: Circulating tumor cells
EMT: Epithelial-to-mesenchymal transition
ECM: Extracellular matrix
ChIP-seq: Chromatin immunoprecipitation and sequencing
H3K27: Histone H3 lysine 27
ROS: Reactive oxygen species
HB: Hemoglobin
SOD: Superoxide dismutase
GPX: Glutathione peroxidase
HDM: High density microporous
scRNA-seq: Single-cell RNA sequencing
t-SNE: T-stochastic neighbor embedding
UMAP: Uniform Manifold Approximation and Projection
NSG: NOD/SCID gamma
DEGs: Differentially expressed genes
LVI: Lymphovascular invasion
PCA: Principal components analysis
HPA: Human Protein Atlas
nTPM: Normalized transcripts per kilobase million
GO: Gene Ontology
DCDMS: Dichlorodimethylsilane
sulpho-SANPAH: N-sulphosuccinimidyl-6-(4'-azido-2'-nitrophenylamino) hexanoate
NS: Non-silencing
UMIs: Unique molecular identifiers
