# Correction: Reprogramming anchorage dependency by adherent‑to‑suspension transition promotes metastatic dissemination

**DOI:** 10.1186/s12943-023-01838-3

**Published:** 2023-08-14

**Authors:** Hyunbin D. Huh, Yujin Sub, Jongwook Oh, Ye Eun Kim, Ju Young Lee, Hwa‑Ryeon Kim, Soyeon Lee, Hannah Lee, Sehyung Pak, Sebastian E. Amos, Danielle Vahala, Jae Hyung Park, Ji Eun Shin, So Yeon Park, Han Sang Kim, Young Hoon Roh, Han‑Woong Lee, Kun‑Liang Guan, Yu Suk Choi, Joon Jeong, Junjeong Choi, Jae‑Seok Roe, Heon Yung Gee, Hyun Woo Park

**Affiliations:** 1 Department of Biochemistry, College of Life Science and Biotechnology, Brain Korea 21 Project, Yonsei University, Seoul, 03722 Republic of Korea; 2 Department of Pharmacology, Graduate School of Medical Science, Yonsei University College of Medicine, Brain Korea 21, Project, Seoul, 03722 Republic of Korea; 3 Cytogen, Seoul, Republic of Korea; 4 School of Human Sciences, University of Western Australia, Crawley, WA 6009 Australia; 5 Yonsei Cancer Center, Division of Medical Oncology, Department of Internal Medicine, Brain Korea 21 Plus Project for Medical Sciences, Severance Biomedical Science Institute, Yonsei University College of Medicine, 03722 Seoul, Republic of Korea; 6 Department of Biotechnology, College of Life Science and Biotechnology, Yonsei University, 03722 Seoul, Republic of Korea; 7 Department of Pharmacology and Moores Cancer Center, University of California San Diego, La Jolla, 92093 CA USA; 8 Departments of Surgery, Gangnam Severance Hospital, Yonsei University College of Medicine, 06273 Seoul, Republic of Korea; 9 College of Pharmacy, Yonsei Institute of Pharmaceutical Sciences, Yonsei University, Incheon, Republic of Korea

**Correction: Mol Cancer 22, 63 (2023)**

10.1186/s12943-023-01753-7.

Following publication of the original article [1], the author reported that the below Methods section needs to be added to the body text.

## Methods

### Bioinformatics of published human cell line RNA-seq data analyses

RNA-seq fastq files were obtained from the ENCODE database. CLC Genomics Workbench 9.5.3 software (Qiagen, Germany) was used to map the reads to the human genome (hg19, build name GRCh37) and to generate gene expression values in the form of normalized transcripts per kilobase million (nTPM). Microarray data were obtained from the Human Protein Atlas (HPA) database in the form of matrices (row – official gene symbol, column – cell line) that included transcripts per million (TPM) values. Each of these data sets were imported into R for further analysis. Then, the data from the HPA were quantile normalized. R v3.6.3 was used to perform principal components and correlation analyses. Hierarchical clustering of these data was performed with the R package Pheatmap (version1.0.12). All cell lines were grouped into adhesion cells verus suspension cells based on their known morphology and anchorage dependency. Differentially expressed genes (DEGs) were selected based on absolute fold changes > 2, absolute TPM differences > 1, and p-values < 0.05, as measured in Gaussian T-tests. Gene Ontology (GO) enrichment analysis was performed with p-values < 0.05 (Benjamini-Hochberg) considered statistically significant. A Gene Set Enrichment Analysis (GSEA) was performed with a random combination number of 1000 and a false discovery rate (FDR) of 0.05 as criteria for significant enrichment. The “.gmt” format data were obtained from the Molecular Signature Database (MSigDB) using GSEA version 4.2.3. The specific gene sets used for these analyses are specified in Table S3. List of all transcription factors (Lambert et al., 2018) were included in the screen for AST factors (Table S4). Candidate AST factors were selected based on their nTPM differences, fold changes, and p-values.

### DNA, shRNA, and CRISPR/Cas9 constructs and AST^TetR^ and Ikzf1^−/−^ cell line generation

Candidate human AST genes were tagged with V5 and FLAG and subcloned into the gateway entry vector pENTR4 vector (Addgene, 17,425 and 17,423). The resulting subcloned vectors were recombined with destination vector pLentiCMV vector (Addgene, 17,293) using LR recombinase (Invitrogen, 1,179,019) to generate lentiviral expression vectors. To generate doxycycline-inducible HEK293A-AST-TetR cells, TetR plasmid (Addgene, 17,492) was infected in HEK293A-AST cells. The shRNA oligonucleotides that specifically targets 4 final AST genes (i.e., IKZF1, NFE2, IRF8 and BTG2) were annealed and subcloned into the pLKO.1 vector using the following target sequences: shRNA-IKZF1, GCTATCAATCATTAAGGTCAT; shRNA-NFE2, CCCATACTCCTATGGCAACAT; shRNA-IRF8, GCCCGCATCATGATTAAAGAA; shRNA-BTG2, CTATGAGGTGTCCTACCGCAT. To generate Ikzf1^−/−^-B16F10 cells, single guide RNAs (sgRNAs) targeting mouse Ikzf1 (sgRNA1, TTTACGAATGCTTGATGCTC; sgRNA2, CAAGGCAGCTCGGCTTTGTC) were cloned into BsmBI-digested Lenti-CRISPR v2 (plasmid #52,961; Addgene). All constructs were verified by sequencing.

### Cell culture

All cells were maintained in an atmosphere of 5% CO_2_ in a 37°C humidified incubator. HEK293A and HEK293T, and B16F10 cells were cultured in DMEM (Hyclone, SH30022.01) and MDA-MB-231, LM2 and SUIT-2 cells were cultured in RPMI (Hyclone, SH30027.01) containing 10% FBS (Hyclone, SV30207.02) and 1% penicillin/streptomycin (Invitrogen, 15,140,122). No cell lines used in this study were found in the database of commonly misidentified cell lines that is maintained by ICLAC and NCBI Biosample. Cells lines were tested and confirmed to be free of mycoplasma. For fibronectin coating, a glass-bottom 6-well culture plate was pre-treated with fibronectin diluted in diH_2_O (ThermoFisher Scientific, 33,016,015) (100 mg/ml) for 30 min. HEK293A-AST^TetR^ cells were seeded onto an untreated glass-bottom 6-well culture plate and a fibronectin treated plate. Cells were left to culture with DMEM for 3 days to ensure < 80% confluency before being treated with doxycycline (Sigma Aldrich, D3072) (10 µg/ml). After 48 h samples were fixed for immunocytochemistry.

### Viral infection

HEK 293T cells were transfected with plasmids encoding pMD2G and psPAX2, along with constructs cloned into lentiviral vector using Polyplus Reagent (Merck) according to the manufacturer’s protocol. Media containing viral particles were harvested at 48 h post-transfection and filtered through a 0.45 mm filter, supplemented with 8 mg/ml polybrene, and used for infection. 24 h after infection, transduced cells were incubated with fresh media for another 24 h and then selected with puromycin or blasticidin.

### Induction and measurement of adherent-to-suspension transition (AST) efficiency

HEK293A cells (5 × 10^5^) were seeded in 6-well culture plates supplemented with media containing viral particles encoding AST candidate genes. 2 days after infection, the transduced cells were trypsinized, reseeded on new plates, and treated with puromycin (4 mg/ml) for selection. The efficiency of AST is quantified by measuring the relative ratio of viable cells between the adherent and suspension cell populations using CellTiter Glo® 2.0 cell viability assay (Promega, G9241).

### Cell viability and ROS detection assay

In vitro cell viability was assessed using the CellTiter Glo® 2.0 cell viability assay (Promega, G9241) and LIVE/DEAD® cell imaging kit (ThermoFisher, R37601) according to the manufacturer’s protocols. For suspension assay, cells were plated on Corning® Costar® Ultra-Low Attachment Multiple Well Plates (Merck, CLS3471). After 24 h, the level of intracellular ROS was measured using the ROS detection kit (ThermoFisher, C10444) according to the manufacturer’s protocol. N-acetyl-L-cysteine (Sigma, A9165) was used as antioxidant agent.

### Immunoblotting analysis

Immunoblotting was performed using a standard protocol. Protein samples were separated by SDS– PAGE and were then transferred onto polyvinylidene fluoride membranes. After blocking with 5% nonfat milk in TBS-T, membranes were incubated with the primary antibody. The phos-tag reagents were purchased from Wako Chemicals (AAL-107), and gels containing phos-tag were prepared according to manufacturer’s instructions. YAP proteins can be separated into multiple bands in the presence of phos-tag depending on differential phosphorylation levels by LATS. Phosphorylated YAP migrates more slowly (upper bands) compared to dephosphorylated YAP (lower bands). The following antibodies were used for western blot analysis: anti-FLAG (Sigma Aldrich, F1804-200MG), anti-V5 (Santa Cruz, sc-271,944), anti-E-cadherin (Cell Signaling, 3195 S), anti-N-cadherin (BD, 610,920), anti-vimentin (Santa Cruz, sc-373,717), anti-GAPDH (Santa Cruz, sc-25,778), anti-IKZF1 (Cell Signaling, 9034 S; Abcam, ab180713), anti-BTG2 (Abcam, ab85051), anti-IRF8 (Abcam, ab28696) and NFE2 (Novus, NBP-82,580), anti-TEAD (Cell Signaling, 13,295), anti-YAP (Cell Signaling, 14,074), anti-TAZ (Cell Signaling, 4883).

### 3D hydrogel encapsulation for AST factor staining

To visualize IKZF1 and V5 tag expression, mock and AST cells were encapsulated in 3D gelatin methacryloyl (GelMA) hydrogels. A 9% weight-to-volume GelMA solution was prepared by dissolving the lyophilized monomer at 37 °C in PBS containing 0.1% weight-to-volume Irgacure-2959 (Sigma-Aldrich, 410896-10G) dissolved in ethanol. The solution was prepared 24 h prior to use, stored at 4 °C overnight and heated to 37 °C in a water bath one hour before use. Prior to encapsulation, #1.5 glass bottomed 6 micro-well plates (Cellvis, P06-20-1.5-N) were cleaned with ozone (Bioforce Nanosciences, PC440) for minutes before the addition of 400 µl/microwell of methacrylate solution comprised of 0.005% 3-(Trimethoxysilyl)-propyl methacrylate (Sigma Aldrich, 440,159) and 3% acetic acid (ThermoFisher Scientific, AJA2-2.5PL) in absolute ethanol (ThermoFisher, AJA214-2.5LPL) for 5 min. This functionalizing solution was replaced with the same volume of absolute ethanol per microwell for 2 min before being left to air dry. Meanwhile, 15 mm diameter circular coverslips (Australian Scientific, 0111550) were treated with one drop per side of Dichlorodimethylsilane (DCDMS) (Sigma Aldrich, 440,272) to facilitate their removal following hydrogel polymerization. HEK293A and SUIT-2 (mock and AST) were separately combined with the GelMA solution at a density of 1 × 104 cells/gels. 50 µl of cell-ladened GelMA was pipetted onto the functionalized surface of each well, immediately covered with a DCDMS-treated coverslip and polymerized with an ultraviolet transilluminator (365 nm) (Fisher Biotech Australia, 2131-2013-1) for 1 min 30 s. The DCDMS-treated coverslip was then removed, and samples were rinsed 3 times with DPBS (ThermoFisher Scientific, 14-190-250) prior to immediate fixation and subsequent immunocytochemistry.

### Immunocytochemistry and imaging of AST factors

Samples are washed three times with DPBS (ThermoFisher Scientific; 14,190,250) before fixation with 4% (w/v) paraformaldehyde (PFA) (Santa Cruz Biotechnology, sc2811692) for 15 min at room temperature (RT). Between each step, samples were washed three times with PBS (ThermoFisher Scientific, 18,912,014) for 5 min with gentle shaking for 3D hydrogel encapsulated samples. Samples were permeabilized with 1% (w/v) Triton X-100 (Sigma Aldrich, X100) in PBS for 15 min before blocking with 5% goat serum (v/v) (Sigma Aldrich, G9023) in PBS for one hour at 37oC. Primary antibodies were diluted 1:100 in 2% bovine serum albumin (BSA) (Sigma Aldrich, A2058) and incubated with the sample for 24 h at 4oC. The following primary antibodies were used for immunocytochemistry: anti-Ikaros (Abcam, ab180713) anti-Vinculin (Abcam, ab129002), anti-V5Tag (Abcam, ab206571). Secondary antibodies were diluted 1:200 in 2% BSA and left to incubate with samples for up to two hours at 37oC. The following secondary antibodies and stains were used for immunocytochemistry: goat anti-rat 488 (Abcam, ab150157), goat anti-rabbit 488 (Abcam, ab150077), goat anti-rabbit 647 (Abcam, ab150079), and rhodamine-phalloidin (Invitrogen, R415). DAPI (Sigma Aldrich, D9542) prepared in a stock solution (5 mg/ml) in DiH2O was diluted in PBS (1:400) and incubated with samples for 15 min at RT. Samples were mounted with SlowFade Diamond Mounting Agent (Thermo Fischer Scientific, S36963) and sealed with nail polish. Samples were imaged on a Nikon A1 confocal microscope with a Plan Apo VC 60x Oil objective immersed in type F oil. For 3D encapsulated cells, a digital zoom of 3x was used during acquisition, lookup tables were scaled equally, and images were cropped to 50 × 50 micron squares in FIJI/ImageJ for display.

### Low-stiffness plates

Polyacrylamide (PA) hydrogels of 10 kPa (5.2% acrylamide/0.3% bis-acrylamide; Bio-Rad, 1,610,140/1,610,142) and 30 kPa (8.8% acrylamide/0.3% bis-acrylamide) were fabricated (Tse & Engler, 2010). Glass bottomed 6 micro-well plates were methacrylated as described above while 25 mm diameter circular coverslips (Grale, CS25100) were treated with DCDMS. PA hydrogel polymerization was initiated by addition of 10 µl of 10% ammonium persulfate (Bio-rad, 1,610,700) and 1 µl N,N,N’,N’- tetramethylethylenediamine (Bio-rad, 1,610,800) into 1 ml of PA solution. The treated microwells were filled with initiated PA solution and covered with a DCDMS-treated coverslip. PA hydrogels were left to polymerize for 20 min before the coverslips were removed, PBS was added to the wells and the plates were sterilized with UV light (302 nm) for 15 min. The PBS was replaced with 2 ml of 1 mg/ml N-sulphosuccinimidyl-6-(4’-azido-2’-nitrophenylamino) hexanoate (sulpho-SANPAH) (ThermoFisher Scientific, 22,589) in pH 8.5 Hepes before UV light (365 nm) activation for 10 min. PA hydrogels were washed three times with pH 8.5 Hepes before being incubated with 2 ml of 25 ug/ml human plasma fibronectin (ThermoFisher Scientific, 33,016,015) overnight at 37 ^o^C.

### Quantitative real-time PCR analysis

Cells were harvested for RNA extraction using the RNeasy Plus mini kit (QIAGEN, 74136). RNA samples were reverse transcribed to complementary DNA (cDNA) using iScript reverse transcriptase (Bio-Rad, 1708891). qRT-PCR was performed using the KAPA SYBR FAST qPCR kit (Kapa Biosystems, KK4605) and the 7300 real-time PCR system (Applied Biosystems). The following primers were used for qPCR : IKZF1, 5’-TTTCAGGGAAGGAAAGCCCC-3’ (forward) and 5’-CTCCGCACATTCTTCCCCAT-3’ (reverse). ; NFE2, 5’-ACAGCTGTCCACTTCAGAGC-3’ (forward) and 5’-TGAGCAGGGGCAGTAAGTTG-3’ (reverse). ; BTG2, 5’-AAACACCACTGGTTTCCCGA-3’ (forward) and 5’-TACAAGACGCAGATGGAGCC-3’ (reverse). ; IRF8, 5’-AGCATGTTCCGGATCCCTTG-3’ (forward) and 5’-CGGTCCGTCACTTCCTCAAA-3’ (reverse). ; KLF1, 5’-ATGACTTCCTCAAGTGGTGGC-3’ (forward) and 5’CCGAGAAGTTGGTGAGGAGGA-3’ (reverse). ; GFI1B, 5’-GGATGCGTACCACTGTGTGA-3’ (forward) and 5’-TCGGACTTCTGGTGGAAACG-3’ (reverse). TAL1, 5’-TGAAGAGGAGACCTTCCCCC-3’ (forward) and 5’-AAAGGCCCCGTTCACATTCT-3’ (reverse). ; SPIB, 5’-CTGTCTGTACCCAGATGGCG-3’ (forward) and 5’-GGTCGAAGGCTTCATAGGGG-3’ (reverse). ; AKNA, 5’-CCCACCCTATCACCAGGGTA-3’ (forward) and 5’-AATGGTGTTCTCGGCCTCAG-3’ (reverse). ; MYB, 5’-ACAGATGGGCAGAAATCGCA-3’ (forward) and 5’-GCTGGCTGGCTTTTGAAGAC-3’ (reverse). ; IKZF3, 5’-GAGAAGGCCCAGCCAATGAA-3’ (forward) and 5’-AGGGATTTCAGGCTCTTCTGC-3’ (reverse). ; GATA1, 5’-CTCCCAAGCTTCGTGGAACT-3’ (forward) and 5’-AGGCGTTGCATAGGTAGTGG-3’ (reverse). ; POU2F2, 5’-AAGTGGCTCAACGATGCAGA-3’ (forward) and 5’-ACGTTTGTCTCGATGCTGGT-3’ (reverse). ; IRF5, 5’-CAGGTGAACAGCTGCCAGTA-3’ (forward) and 5’-CCAGGCCTTGAAGATGGTGT-3’ (reverse). ; RHOXF2, 5’-GGAACCTGGGCAGCAGTAT-3’ (forward) and 5’-GAATGGCTGTGGTCCCAGAA-3’ (reverse). ; BCL11A, 5’-ATGCACAAATCGTCCCCCAT-3’ (forward) and 5’-GGGTCGTTCTCGCTCTTGAA-3’ (reverse). ; KLF2, 5’-AGACCTACACCAAGAGTTCGCATC-3’ (forward) and 5’-CATGTGCCGTTTCATGTGCAGC-3’ (reverse). ; TCF7, 5’-CAAGCAGAGTCCAAGGCAGA-3’ (forward) and 5’-AGGATCTGGTTGATGGCAGC-3’ (reverse). ; SPI1, 5’-GCGTGCAAAATGGAAGGGTT-3’ (forward) and 5’-GCTATGGCTCTCCCCATCAC-3’ (reverse). ; EAF2, 5’-AATAGCGCAGCGGGATTCTC-3’ (forward) and 5’-TCATAGCGCACAGTGTGGA-3’ (reverse). ; ACTB, 5’-GCCGACAGGATGCAGAAGGAGATCA-3’(forward) and 5’-AAGCATTTGCGGTGGACGATGGA-3’(reverse).

### siRNA transfection

Cells were transfected with transfection mix (100 µl of Opti-MEM™ medium (ThermoFisher, #31,985,070), 12 ml RNAi Max (Invitrogen, 13,778,150), and 2 µl of 20 µM siRNA). The following siRNA were used for transfection: non-silencing (NS) siRNA (Dharmacon siGENOME non-targeting control pool #D-001206-13-200), siHBA1 (Dharmacon siGENOME SMARTPool #M-011114-01-0010), siIKZF1 (Dharmacon siGENOME SMARTPool #M-019092-01-0010), siNFE2 (Dharmacon siGENOME SMARTPool #M-010049-00-0010), siIRF8 (Dharmacon siGENOME SMARTPool #M-011699-01-0010), siBTG2 (Dharmacon siGENOME SMARTPool #M-012308-00-0010), and siPOU2F2 (Dharmacon siGENOME SMARTPool #M-019690-01-0010).

**Specimens of*de novo*metastatic breast cancer**.

From November 2021, three patients diagnosed with stage IV breast cancer with no prior anticancer treatments were recruited to this study. Fresh primary tumor tissues and 20 ml blood were obtained from each patient for scRNA-seq experiment. Subtypes of patient 1 and patient 2 are Her2-positive breast cancer and patient 3 is luminal B breast cancer. Patient 1 was a 44-year-old female with multiple liver and bone metastases. Patient 2 was a 52-year-old female with multiple lung and bone metastasis. Patient 3 was a 72-year-old female who developed lung metastasis 8 years after initial surgery. In this patient, cells isolated from primary tumor did not pass the quality control for library preparation; therefore, only blood sample was further processed. Ethical approval for the use of human subjects was obtained from the Institutional Review Board of Gangnam Severance Hospital, Yonsei University, Seoul, Republic of Korea (3-2021-0236) in compliance with the ethical guidelines of the 1975 Declaration of Helsinki, and informed written consent was obtained from each patient.

### Orthotopic mouse model experiments

The animal experiment protocols were reviewed and approved by the Yonsei University Institutional Animal Care and Use Committee (IACUC). All mice were handled following the Guidelines for the Care and Use of Laboratory Animals. NOD SCID Gamma (NSG) mice were purchased from JA bio. For orthotopic injection experiments, 1 × 10^6^ of LM2 cells were injected orthotopically into the fat-pad of 7- week-old female NSG mice. For in vivo treatment with pomalidomide or lenalidomide, NSG mice were injected IP every 3 days with 40 µL containing 10 mg/kg of pomalidomide or lenalidomide diluted in DMSO. At the end point (week 3), the mice were sacrificed, and their lungs were rapidly excised for ex vivo bioluminescence imaging using In vivo imaging VISQUE InVivo Smart-LF (Viewworks, BI2004S001). All in vivo xenograft experiments were carried out using mice which received identical treatment. After fat pad injection, mice were randomly divided into different groups for treatment with shRNA, pomalidomide, or lenalidomide. The NSG mice used in these experiments all had identical genotype and genetic background. C57BL/6 N mice were purchased from Orient Bio. For the B16F10 cell footpad implantation model, 5 × 10^5^ GFP-positive B16F10 melanoma cells were implanted subcutaneously into the footpads of 7-week-old male mice. For in vivo treatment with lenalidomide, C57BL/6 N mice were IP-injected every day with 40 µL of 10 mg/kg of lenalidomide diluted in DMSO. Mice were sacrificed after week 8. None of the experiments performed in this study surpassed the size limit of the tumors (the volume did not exceed 2 cm^3^).

### CTC isolation and analysis

Blood samples were collected in acid citrate dextrose solution A tubes (BD Vacutainer®). 5mM EDTA was added to blood samples and rotated with multi mixer (Seoulin bioscience, SLRM-3). The samples were processed to enrich CTCs using a CTC isolation kit (Cytogen, CIKW10) and SMART BIOPSY™ Cell Isolator (Cytogen, CIS030). The presence of CTCs was verified by multi-immunofluorescence staining after making slides with Cytospin (ThermoFisher Scientific, Cytospin 4). The nucleic acid dye 4’6-diamidino-2-phenylindole (DAPI) was used to label the nucleated cells and CD45 was used to identify leukocytes. CTCs were defined as DAPI-positive, epithelial cell adhesion molecule (EpCAM)-positive, and CD45-negative. Cells were stained and analyzed with each marker using a SMART BIOPSY™ Cell Image Analyzer (Cytogen, CIA030) and its image analyzing software. For mouse samples, after scRNA-seq, (1) puromycin-resistant (puroR) gene and (2) transcriptome aligned to the human reference genome above 80% identity was used to clarify that isolated cells are pure LM2-derived cancer cells. For the melanoma mouse experiments, 0.8 ml of blood was collected through cardiac puncture and processed immediately. Samples were stained only for CD45, as cancer cells were identified on the basis of GFP expression. The GFP-positive tumor cells were sorted using a FACS Aria II (BD Bioscience).

### Breast tissue dissociation

Tumor tissues of sizes ranging from 0.05 to 0.2 g were dissociated in 2.5 mL enzyme mix, while those ranging from 0.2 to 1.0 g were dissociated in 5 mL enzyme mix. The tumors were cut into small pieces of 2–4 mm pieces, then were transferred into the gentleMACS C-tubes (Miltenyi Biotec, 130-096-334) containing the enzyme mix. C-tubes were attached to the sleeve of the gentleMACS Dissociator (Miltenyi Biotec, 130-093-235), and Dissociator was run through the gentleMACS program. After completion of the program, C-tube detached from the gentleMACS Dissociator and incubated for 30 min at 37 °C under continuous rotation. After the incubation, the Gentle MACS Dissociator program was repeated 3 more times. The sample in the C-tubes were added to 5ml of media and filtered subsequently through a 70 mm strainer and a 20 mm strainer. After 5 min of centrifugation at 800 g, the supernatant was carefully removed from the pellet. After adding 1 ml of RBC lysis buffer (Invitrogen, 00-4333-57), the samples were mixed by pipetting and incubated at RT for 3 min after. After 3 min of centrifugation at 1200 rpm, the supernatant was carefully removed. The pellet was mixed with 500 ml of PBS and then moved to an ep-tube for measuring cell number and viability.

### Melanoma primary tumor and lung metastasis tissue dissociation

GFP-positive B16F10 cells were isolated from the primary sites and lungs. Primary tumor and metastasized lung tissues were digested in 5 ml of enzyme buffer containing 0.2 mg/ml collagenase type-II (Worthington), 0.1 mg/ml DNase I (Roche) and 0.8 mg/ml dispase (Gibco) at 37 °C for 30 min. After complete digestion, the resulting cell suspension was filtered through a 40 μm-sized cell strainer and washed. Then, RBC lysis was performed in suspension in ACK lysis buffer (Gibco) for 3 min at RT.

### Hematoxylin-eosin staining, immunohistochemistry, and quantification of tumor burden

Routine hematoxylin-eosin staining (Muto pure chemical, 2002-2) was performed on 5-µm-thick formaldehyde fixed paraffin-embedded tissue sections. All immunohistochemistry was performed with formalin-fixed, paraffin-embedded tissue sections using an automatic immunohistochemistry staining device (Benchmark XT, Ventana Medical System). The 5-µm-thick formaldehyde-fixed paraffin-embedded tissue sections were conveyed onto adhesive slides and dried at 62 °C for 30 min. H-E staining was performed with standard heat epitope retrieval for 30 min in EDTA at pH 8.0 (Abcam, ab15575020) in the autostainer. The samples were then incubated with the following primary antibodies: AST related markers IKZF1 (Abcam, ab180713), NFE2 (Abcam, ab140598), IRF8 (Merck, HPA002531), and BTG2 (Abcam, ab8051). After washing, the sections were incubated with biotinylated anti-mouse immunoglobulins, peroxidase-labeled streptavidin (DakoCytomation, K0609), and 3,30-diaminobenzidine. Negative control samples were processed without primary antibodies. Positive control tissues were used according to the manufacturer’s recommendations, and the slides were counterstained with (Muto pure chemicals, 2002-2) Harris hematoxylin. All immunohistochemical markers were visualized by light microscopy. Nuclear expression of each marker was considered positive in this study. Collection of human breast cancer samples were obtained from consenting patients in accordance with guidelines and regulations approved by the Institutional Review Board of Gangnam Severance Hospital, Yonsei University Health System (3-2021-0236). Mouse lung samples were fixed in 10% formaldehyde and embedded in paraffin. Whole lungs were cut into 5 μm sections. All the sections for each lung were scanned and quantified by a pathologist, using ImageJ Analyze function.

### Generation of scRNA-seq data from primary and metastatic tumor cells and CTCs

Single cells were sorted on HDM chips (Smart BiopsyTM Cell Isolator, CIS 030) to enrich cancer cells harvested from human and mouse breast cancer tissue and blood. Single-cell RNA sequencing libraries were created using the Chromium Single Cell 3’ Library, Gel Bead & Multiplex kit and Chip kit (10X Genomics) with a target of 10,000 cells per library according to manufacturer’s suggestions. This droplet-based system uses barcodes (1 for each cell) and unique molecular identifiers (UMIs, 1 for each unique transcript) to obtain a unique 3’-mRNA gene expression profile from every captured cell. All samples were sequenced on an Illumina HiSeq4000. For melanoma mouse experiments, pooled mouse (N = 7) primary, CTC, and lung metastatic tumor cells were used. Single-cell RNA sequencing libraries were prepared using Chromium Next GEM Single Cell 3’ v3.1 Cellplex Kit with multiplexing oligo from 10X Genomics. Cells were labeled with oligo-conjugated lipid tags and pooled in groups of 3 samples before loading on a 10X chip with a target of 10,000 cells. cDNA library quality was determined using an Agilent Bioanalyzer. The generated libraries were pooled targeting 80% gene expression libraries and 20% cell multiplexing libraries. Paired-end sequencing was performed using NovaSeq 5000/6000 S1 Rgt Kit v1.5 (200 cycles) on a NovaSeq600 (Illunmina) sequencer.

### scRNA-seq analysis of primary tumor cells and CTCs

The Illumina outputs of human and mouse samples were mapped to the human reference genome (GRCh38) using Cell Ranger 6.0.0 (10X Genomics). Gene-wise read counts for genes with a least 500 reads were exported from Cell Ranger to the Matrix Market format and imported into R with Seurat’s Read10X function. An average of > 135 million reads and > 13,000 cells were obtained for each sample. Each 10X library was individually checked for quality, and the cells were filtered to ensure good gene coverage, a consistent range of read counts, and few mitochondrial reads. At least 500 detected genes were generally required for each cell, although the lower limit was reduced to 200 or 300 for some libraries. No more than 10% mitochondrial reads were allowed per cell, although the upper limit was increased as high as 65% and 92% for cells derived from primary tumors and blood, respectively. Cells with exceptionally high read counts or genes detected were also filtered to minimize the occurrence of doublets. To prevent mouse-derived cells from contaminating the results, cells with more than 20% unmapped reads were also filtered. After this quality filtering, 15,542 cells from primary sites (1,433 cells from patient 1; 14,109 cells from patient 3) and 55,580 cells from PBMC (6,733 cells from patient 1; 23,153 cells from patient 2; 25,694 cells from patient 3) were subjected to further analysis for *de novo* metastatic breast cancer human samples. For mouse samples, puromycin-resistant (puroR) gene and transcriptome aligned to the human reference genome above 80% identity was used to clarify that isolated cells are pure LM2-derived cancer cells. 5,511 primary tumor cells and 3,006 cells isolated from blood samples were subjected to further analysis. Statistical analyses of the 10x data were conducted using the Seurat software package (v4.1.0) (Seurat et al., 2019) for R.

Samples were combined via merging in Seurat. Cell clusters were identified using the default Louvain clustering algorithm implemented in Seurat. Default Seurat function settings were used and 1:10 principal component dimensions were used for all dimension reduction and integration steps. The cluster resolutions were set to 1.0 unless otherwise stated. The RunUMAP random seed was set to 1,000 to ensure reproducibility. Marker genes for cell clusters were identified using Seurat’s FindAllMarkers function with the default settings. For single-cell RNA sequencing of B16F10-derived melanoma, we used the melanocyte-specific marker genes, *Pmel, Mlana, Tryp1, Sox10* and *Cspg4*.

### Chromatin immunoprecipitation (ChIP) assay

5 × 10^6^ single trypsinized HEK293A-Mock and 4AST cells were cross-linked for 15 min with 1% formaldehyde, followed by quenching with 0.125 M glycine for 10 min at room temperature, and washed with PBS. The cell pellets were lysed with 200 µl of cell lysis buffer [10 mM Tris-Cl pH 8.0, 10 mM NaCl, 0.2% NP-40], with protease inhibitor (cOmplete™ Protease Inhibitor Cocktail, Roche, 11,697,498,001) and 1 mM DTT, and incubated for 10 min on the ice. Chromatin was isolated by centrifuge at 7,400 rpm for 30 s. The pellet was gently resuspended in 200 µl of nuclei lysis buffer [50 mM Tris-Cl pH 8.0, 10 mM EDTA, 1% SDS], with protease inhibitor and 1 mM DTT. Chromatin lysate was sonicated for 10 cycles (30 s on/ 30 s off). The sonicated chromatin mixture was incubated for 1 h with 10 µg of rabbit IgG, and 10 µl of Protein A magnetic beads (Invitrogen, 10,001) for pre-clearing. Immunoprecipitation was conducted with 1 ml of pre-cleared chromatin, 1 µg of the H3K27ac or H3K27me3 antibody (Abcam, ab4729; Millipore, 07-449), and 10 µl of Protein A magnetic beads overnight at 4 °C rotator. Next day, the immunocomplexes were washed with IP Wash I Buffer, second wash twice with High salt buffer, third wash once with IP Wash II buffer, and final wash twice with TE (pH 8.0). The washed immunocomplexes were eluted with 200 µl of elution buffer [1% SDS and 0.1 M NaHCO_3_] for 30 min at 45 °C thermomixer 1,000 rpm. The elute was de-crosslinked with RNase A (1 µg/µl) and 0.25 M NaCl, and incubated overnight at 65 °C water bath. The next day, after 2 h incubation of Proteinase K (NEB, P8107S), the immunoprecipitated DNA was purified with a QIAquick PCR purification kit (QIAGEN, 28,106) in 50 µl of EB (elution buffer).

### ChIP-seq library construction

ChIP-seq libraries were constructed using 40 µl of purified ChIP DNA and NEXTflexTM ChIP-seq kit (PerkinElmer, NOVA-5143-02) according to the manufacturer’s instructions. Briefly, ChIP DNA was end-repaired and size-selected (250–300 bp) using AMPure XP beads (Beckman, A63881). Other procedures from adenylation to PCR amplification were done according to ChIP-seq library construction steps. The quality of ChIP-seq libraries was determined by Bioanalyzer using the High Sensitivity chip (Agilent), and the average size of ChIP-seq libraries ranged from 250 to 350 bp. For multiplexing, equal molar quantities of libraries were combined by considering sequencing depth per sample (20 to 40 million reads per library). ChIP-seq libraries were sequenced using an Illumina NextSeq platform with single-end reads of 76 bases.

### Bioinformatic analyses of ChIP-seq

(1) Alignment and visualizing of ChIP-seq: ChIP-seq raw reads were mapped to the reference human genome assembly hg19 using Bowtie2, and duplicated reads were removed using SAMtools. For visualizing with UCSC genome browser, the makeBigWig tool (in HOMER suite) was used to generate bigWig files.

(2) Identification and annotation of 425 4AST LOSS regions: To define 4AST LOSS regions, HOMER suite was used to identify peaks from each H3K27ac ChIP-seq dataset and combined to identify total union H3K27ac peaks. If the fold changes of HEK293A-Mock versus HEK293A-4AST was greater than four (log2 fold change > 2), they were assigned as 4AST LOSS regions (n = 425). To annotate 4AST LOSS regions, the annotatePeaks tool was used to determine whether a peak was in the TSS (transcription start site), TTS (transcription termination site), exon (Coding), 5’ UTR exon, 3’ UTR exon, intronic, or intergenic. In this study, 5’ UTR and 3’ UTR exons were unified as exon.

(3) Motif enrichment in 4AST LOSS region: To find enriched motifs in 4AST LOSS regions, genomic sequences were further analyzed with TRAP algorithm to identify enriched motifs, compared to JASPAR vertebrates with mouse promoters as the control, and Benjamini-Hochberg as the correction.

### Statistical analysis

All quantitative data were obtained from at least three independent biological replicates. Data are presented as means ± standard deviation (s.d.) unless otherwise noted in the Fig. legends. Statistical differences between two groups were examined using two-tailed, unpaired Student’s t-test and one-way analysis of variance (ANOVA) with Bonferroni corrections for multiple comparisons. Statistical tests were performed using the GraphPad Prism 9.0 software (GraphPad Software, CA, USA). Two-sided p-values of less than 0.05 were considered significant. No statistical methods were used to predetermine sample size. Sample size was based on previous experience with experimental variability. Blinding was performed wherever possible during all sample analysis by coding sample identity during data collection and having analysis performed by an observer without knowledge of or access to experimental condition.
