# Supplementary figures and images for: Reprogramming anchorage dependency by adherent-to-suspension transition promotes metastatic dissemination
Source: Mol Cancer. 2023 Mar 30;22:63. doi: 10.1186/s12943-023-01753-7 (PMC10061822; doi:10.1186/s12943-023-01753-7)

**A**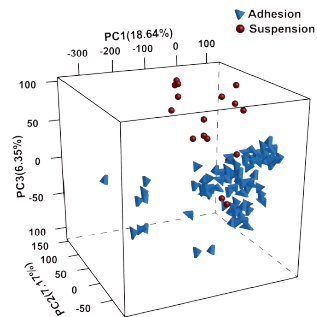**B**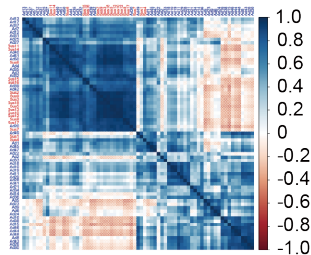**C**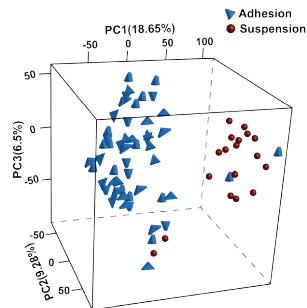**D**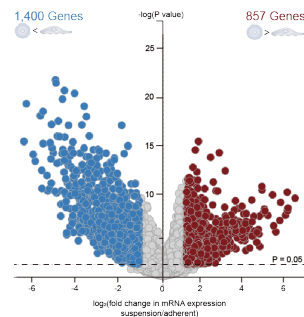**E**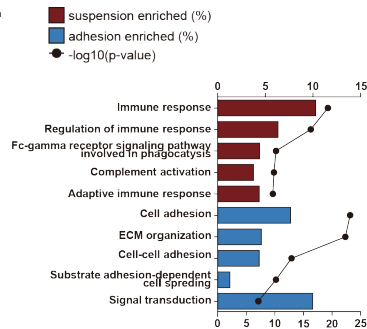**F**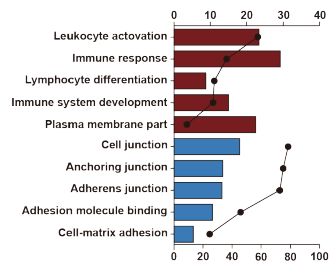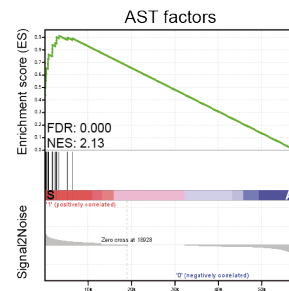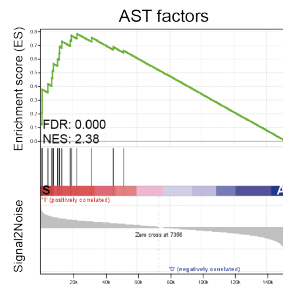

Supplement: Supplementary file 2 — Additional file 2: Figure S1. Analysis of transcriptional expression patterns in adhesion or suspension cells. [file 12943_2023_1753_MOESM2_ESM.pdf]

**A**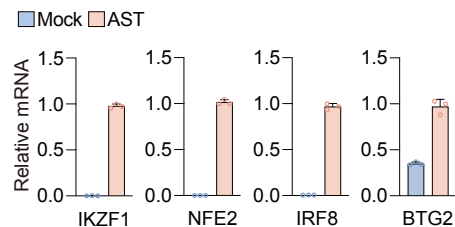**B**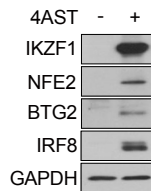**C**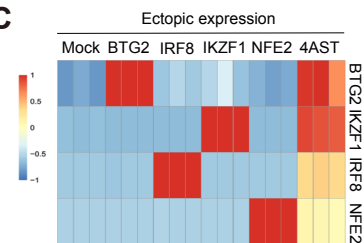**D**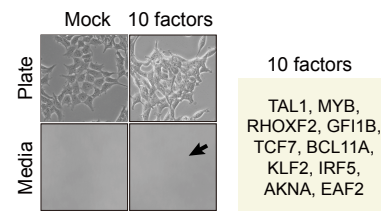**E**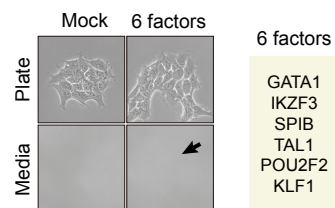**F**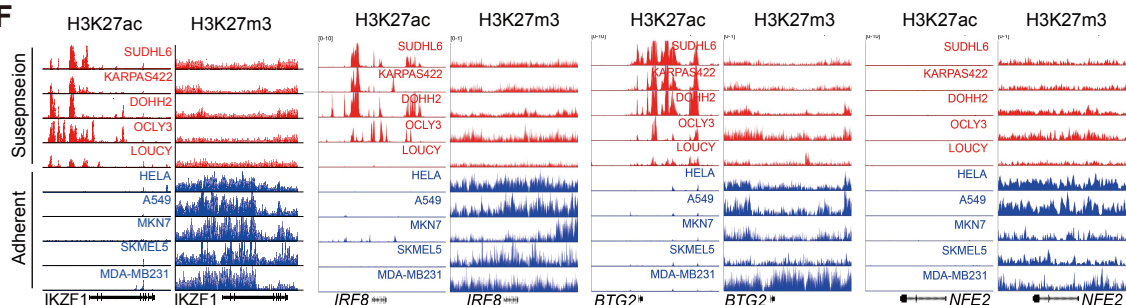**G**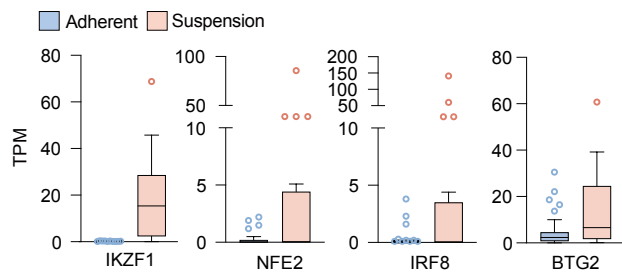**H**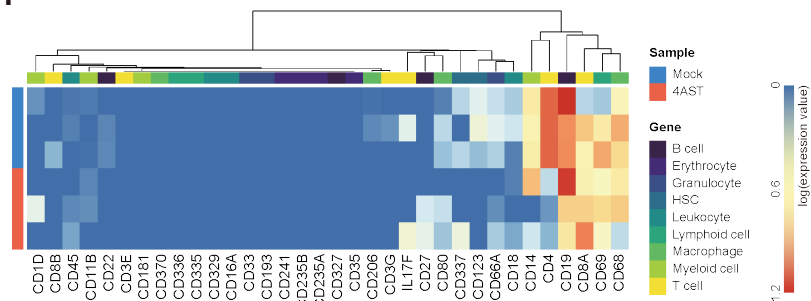

Supplement: Supplementary file 3 — Additional file 3: Figure S2. AST factors evoke cell detachment in the absence of lineage differentiation. [file 12943_2023_1753_MOESM3_ESM.pdf]

**A**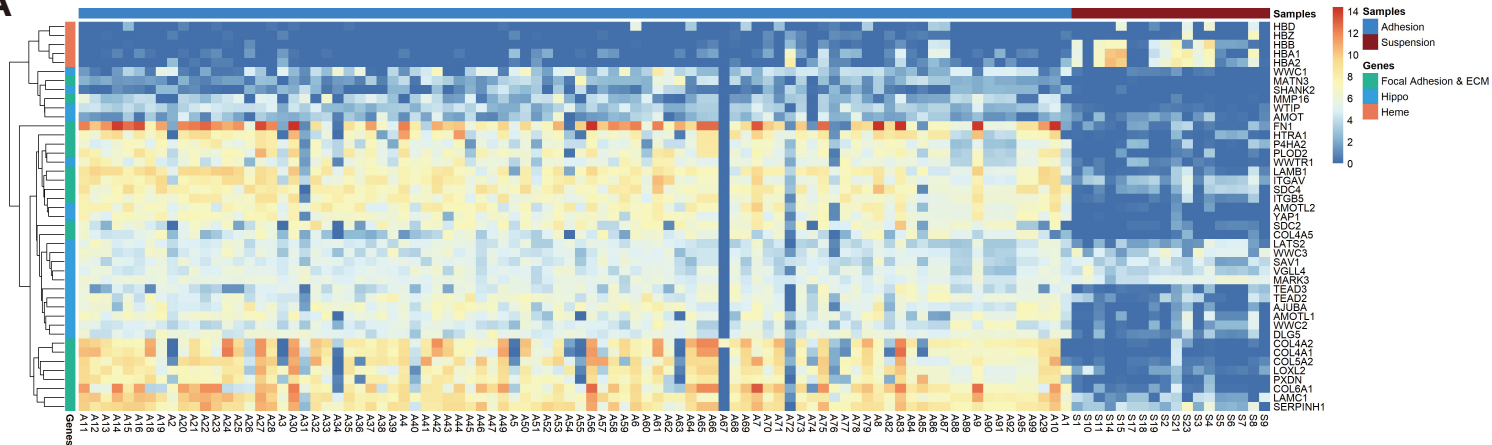**B**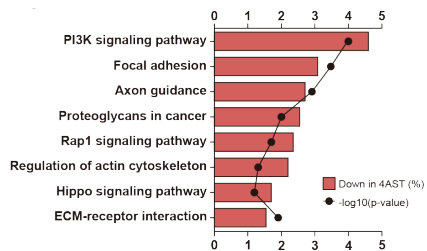**C**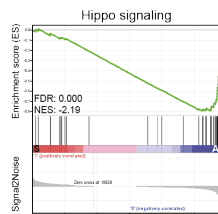**D**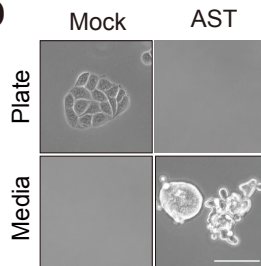**E**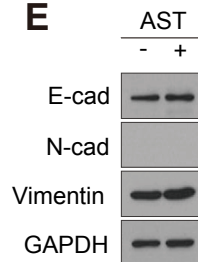**F**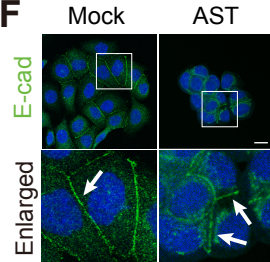

Supplement: Supplementary file 4 — Additional file 4: Figure S3. Gene signatures and mechanisms of the adherent-to-suspension transition. [file 12943_2023_1753_MOESM4_ESM.pdf]

A

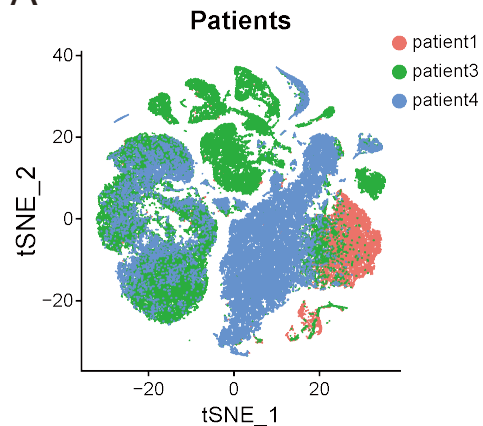

B

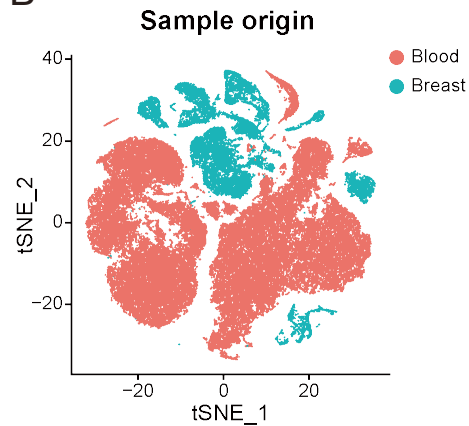

C

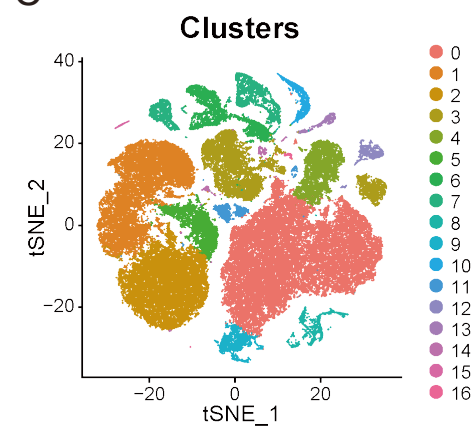

D

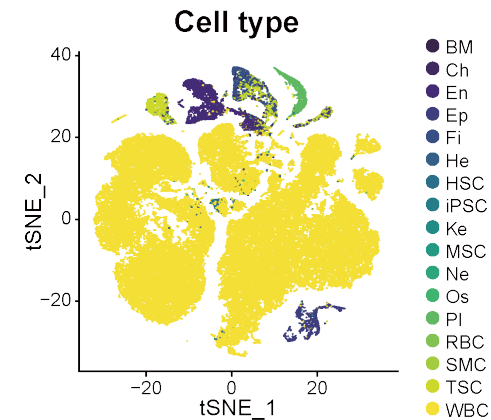

E

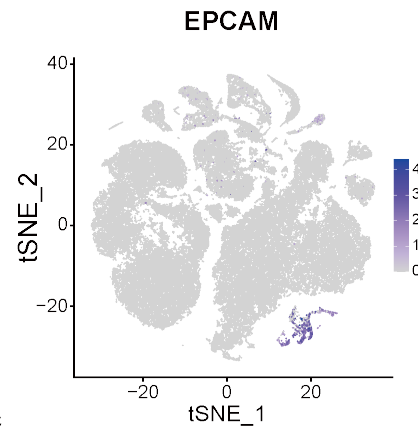

F

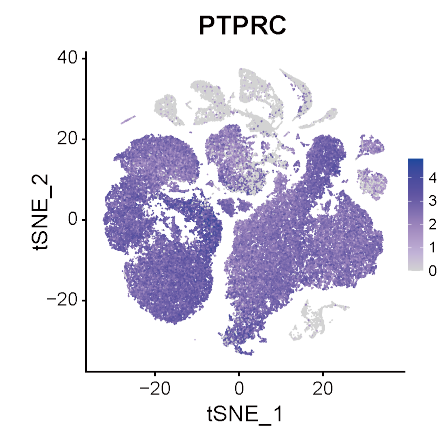

Supplement: Supplementary file 5 — Additional file 5: Figure S4. scRNA-seq analysis of primary tumors and blood cells from de novo metastatic breast cancer patients. [file 12943_2023_1753_MOESM5_ESM.pdf]

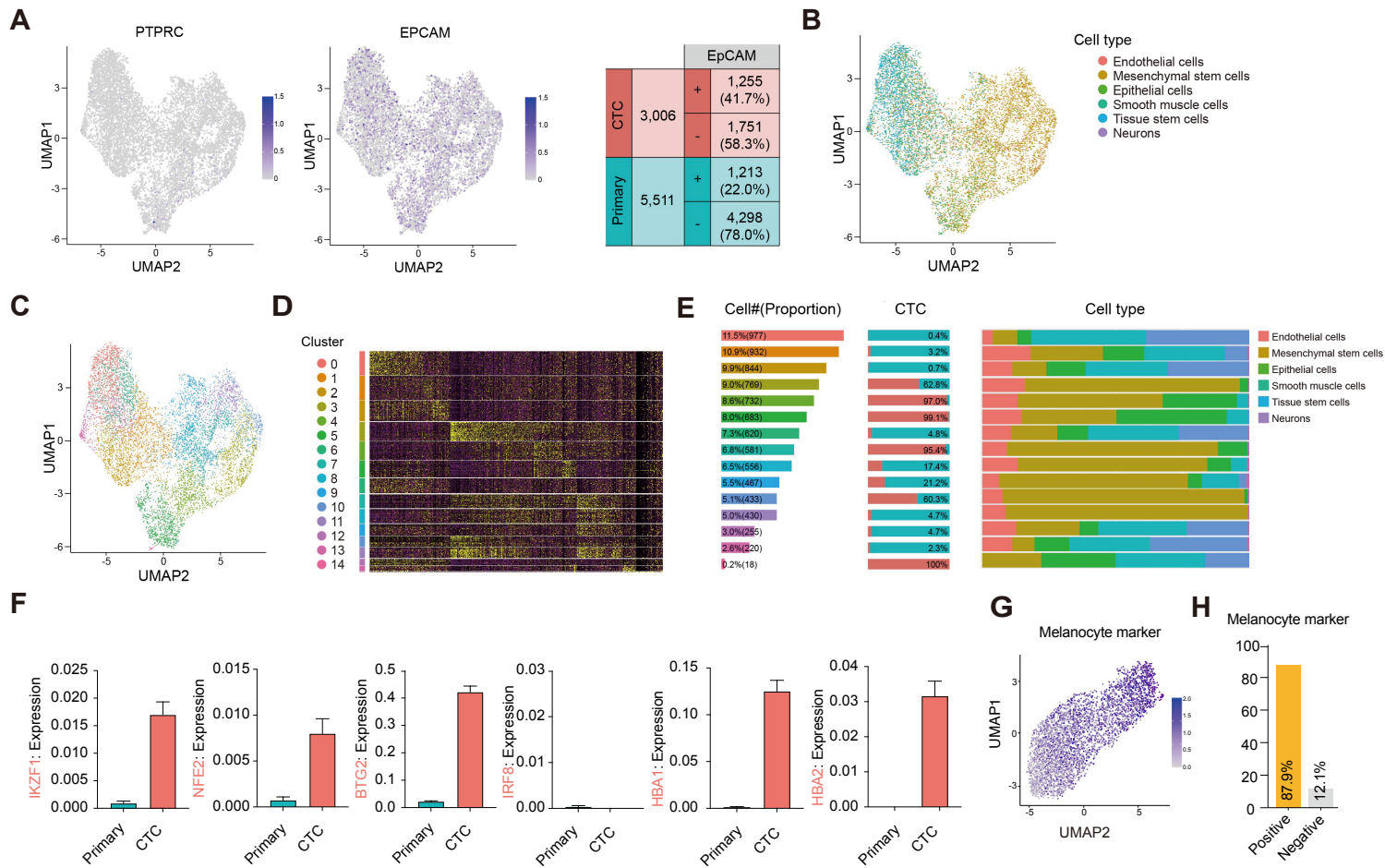

Supplement: Supplementary file 7 — Additional file 7: Figure S6. scRNA-seq analysis of primary tumor and CTCs from breast cancer and melanoma xenograft mouse model. [file 12943_2023_1753_MOESM7_ESM.pdf]

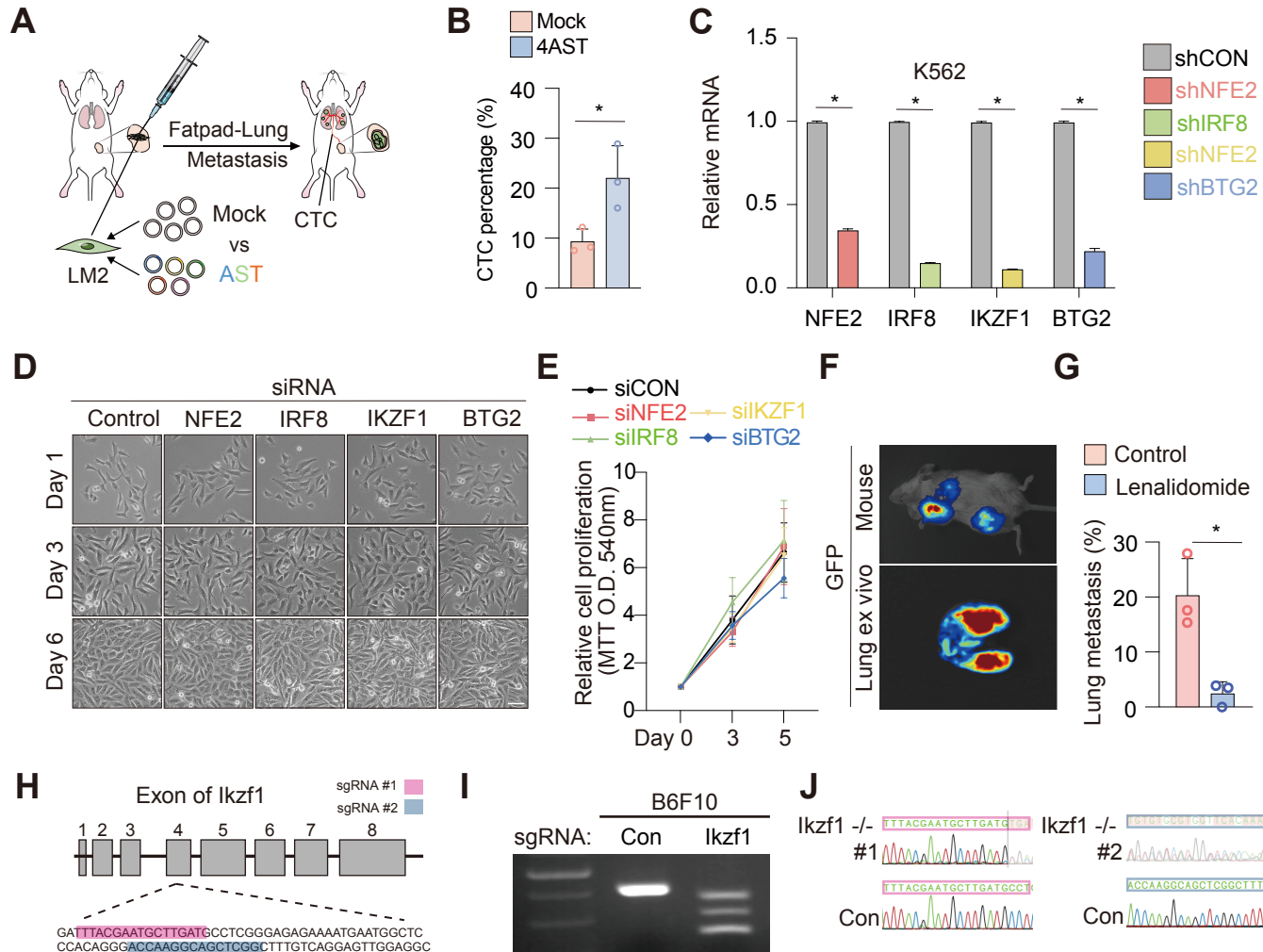

Supplement: Supplementary file 8 — Additional file 8: Figure S7. Effect of AST factor inhibition in cancer cell proliferation and dissemination. [file 12943_2023_1753_MOESM8_ESM.pdf]
