# Supplementary material for: Reprogramming anchorage dependency by adherent-to-suspension transition promotes metastatic dissemination
Source: Mol Cancer. 2023 Mar 30;22:63. doi: 10.1186/s12943-023-01753-7 (PMC10061822; doi:10.1186/s12943-023-01753-7)
Supplement: Supplementary file 9 — Additional file 9: Table S1. RNA sequencing data of adhesion and suspension cells used in this study from Gene Expression Omnibus (GEO). [file 12943_2023_1753_MOESM9_ESM.pdf]

Table S1. RNA sequencing data of adhesion and suspension cells used in this study from Gene Expression Omnibus (GEO)

Analyzed_Samples								
Group	Data Source	Accession	Sample(paired ended/single ended)		labeling	Biosample Type	Cell line	Origin
Adhesion	GEO	GSM958749	ENCFF000DKT	ENCFF000DKZ	A1	Cell line	HCT116	Colorectal Carcinoma
Adhesion	GEO	GSM958749	ENCFF000DKU	ENCFF000DLA	A2	Cell line	HCT116	Colorectal Carcinoma
Adhesion	GEO	GSM958749	ENCFF000DKV	ENCFF000DLB	A3	Cell line	HCT116	Colorectal Carcinoma
Adhesion	GEO	GSM958744	ENCFF000DRT	ENCFF000DRX	A4	Primary Cell		Skeletal muscle myoblast
Adhesion	GEO	GSM958745	ENCFF000EBG	ENCFF000EBN	A5	Cell line	MCF7	Breast Adenocarcinoma
Adhesion	GEO	GSE78585	ENCFF000ENZ	ENCFF000EOA	A6	Cell line	AG04450	Lung fibroblast
Adhesion	GEO	GSE78606	ENCFF000GBQ	ENCFF000GBU	A7	Primary Cell		Hair follicle dermal papilla cell
Adhesion	GEO	GSE78616	ENCFF000GEQ	ENCFF000GET	A8	Primary Cell		Mammary epithelial cell
Adhesion	GEO	GSE90273	ENCFF000GGZ	ENCFF000GHA	A9	Primary Cell		Mesenchymal stem cell of the bone marrow
Adhesion	GEO	GSE78608	ENCFF000GKC	ENCFF000GKF	A10	Primary Cell		Osteoblast
Adhesion	GEO	GSE78604	ENCFF000GKZ	ENCFF000GLH	A11	Primary Cell		Placental Pericyte
Adhesion	GEO	GSE78609	ENCFF000GNB	ENCFF000GNG	A12	Primary Cell		Endothelial cell
Adhesion	GEO	GSE78588	ENCFF000GSA	ENCFF000GTA	A13	Primary Cell		Endothelial cell of umbilical vein
Adhesion	GEO	GSE78615	ENCFF000GYE	ENCFF000GYW	A14	Primary Cell		Subcutaneous white pre-adipocyte
Adhesion	GEO	GSE90257	ENCFF000HAZ	ENCFF000HBG	A15	Cell line	IMR-90	Lung fibroblast
Adhesion	GEO	GSE78611	ENCFF000ILQ	ENCFF000ILS	A16	Primary Cell		Skeletal muscle myosatellite cells
Adhesion	GEO	GSE78536	ENCFF001QZF	ENCFF001QZG	A18	Primary Cell		Dermis blood vessel endothelial cell
Adhesion	GEO	GSE78539	ENCFF001QZH	ENCFF001QZI	A19	Primary Cell		Nasal cavity respiratory epithelium epithelial cell
Adhesion	GEO	GSE78546	ENCFF001QZL	ENCFF001QZM	A20	Primary Cell		Smooth muscle cell of trachea
Adhesion	GEO	GSE78528	ENCFF001QZP	ENCFF001QZQ	A21	Primary Cell		Aortic smooth muscle cell
Adhesion	GEO	GSE78534	ENCFF001QZX	ENCFF001QZY	A22	Primary Cell		Smooth muscle cell of the coronary artery
Adhesion	GEO	GSE78532	ENCFF001RAF	ENCFF001RAG	A23	Primary Cell		Bronchial smooth muscle cell
Adhesion	GEO	GSE78529	ENCFF001RAH	ENCFF001RAI	A24	Primary Cell		Bladder microvasvular endothelial cell
Adhesion	GEO	GSE78533	ENCFF001RBJ	ENCFF001RBK	A25	Primary Cell		Endothelial cell of coronary artery
Adhesion	GEO	GSE78531	ENCFF001RBN	ENCFF001RBO	A26	Primary Cell		Bronchial epithelial cell
Adhesion	GEO	GSE78548	ENCFF001RBX	ENCFF001RBY	A27	Primary Cell		Smooth muscle cell of the umbilical artery
Adhesion	GEO	GSE78549	ENCFF001RCB	ENCFF001RCC	A28	Primary Cell		Uterine smooth muscle cell
Adhesion	GEO	GSE78540	ENCFF001RCD	ENCFF001RCE	A29	Primary Cell		Pulmonary artery endothelial cell
Adhesion	GEO	GSE78541	ENCFF001RDN	ENCFF001RDO	A30	Primary Cell		Smooth muscle cell of the pulmonary artery
Adhesion	GEO	GSE78640	ENCFF002BEF	ENCFF002BEG	A31	Invitro differentiated cell	neural cell originated from H1	Human embryonic stem cell line
Adhesion	GEO	GSE78676	ENCFF002DKO	ENCFF002DKS	A32	Cell line	SJSA1	Ostesarcoma fibroblast
Adhesion	GEO	GSE78652	ENCFF002DHT	ENCFF002DHU	A33	Cell line	A375	Malignant melanoma epithelial cell
Adhesion	GEO	GSE78684	ENCFF002DKW	ENCFF002DLA	A34	Cell line	HT-29	Colorectal adenocarcinoma
Adhesion	GEO	GSE78666	ENCFF002DLN	ENCFF002DLO	A35	Cell line	M059J	Malignant Glioblastoma
Adhesion	GEO	GSE78653	ENCFF002DLP	ENCFF002DLQ	A36	Cell line	HT1080	Fibrosarcoma
Adhesion	GEO	GSE78643	ENCFF002DLX	ENCFF002DLY	A37	Cell line	RPMI7951	Malignant melanoma epithelial cell
Adhesion	GEO	GSE78655	ENCFF002DMB	ENCFF002DMC	A38	Cell line	SJCRH30	Rhabdomyosarcoma fibroblast
Adhesion	GEO	GSE78651	ENCFF002DMJ	ENCFF002DMK	A39	Cell line	H7	Human embryonic stem cell line
Adhesion	GEO	GSE78664	ENCFF002DMF	ENCFF002DMG	A40	Cell line	SK-MEL-5	Malignant melanocyte
Adhesion	GEO	GSE78657	ENCFF002DMR	ENCFF002DMS	A41	Cell line	A172	Glioblastoma
Adhesion	GEO	GSE78647	ENCFF109IUU	ENCFF322VHJ	A42	Primary Cell		Kidney epithelial cell
Adhesion	GEO	GSE78680	ENCFF126HPJ	ENCFF876WTO	A43	Primary Cell		Endometrial microvascular endothelial cells
Adhesion	GEO	GSE78677	ENCFF193NKV	ENCFF700EUG	A44	Invitro differentiated cell	myocyte originated from LHCN-M2	Pectoralis major muscle tissue, satellite cells
Adhesion	GEO	GSM1112817	ENCFF194PWT	ENCFF828ZHU/	A45	Invitro differentiated cell	ectodermal cell originated from HUES64	Human embryonic stem cell line with a normal 46XY karyotype
Adhesion	GEO	GSE78685	ENCFF250OAD	ENCFF952UDX	A46	Cell line	MG63	Osteosarcoma
Adhesion	GEO	GSE78648	ENCFF341UDE	ENCFF420HRW	A47	Cell line	PC-3	Prostate adenocarcinoma
Adhesion	GEO	GSE88167	ENCFF197MZY	ENCFF306XSY	A48	Invitro differentiated cell	cardiac muscle cell originated from RUES2	Human embryonic stem cell line
Adhesion	GEO	GSM915323	ENCFF378MAM	ENCFF663RCH	A49	Invitro differentiated cell	mesenchymal stem cell originated from H1	Human embryonic stem cell line
Adhesion	GEO	GSE88049	ENCFF479JTM	ENCFF671HJW	A50	Primary Cell		Epithelial cell of proximal tubule
Adhesion	GEO	GSM915328	ENCFF039JYG	ENCFF565ZQD	A51	Cell line	H1	Human embryonic stem cell line
Adhesion	GEO	GSE78681	ENCFF288QUM	ENCFF594SZG	A52	Cell line	H4	Human embryonic stem cell line
Adhesion	GEO	GSE78679	ENCFF644RGE	ENCFF653EZE	A53	Invitro differentiated cell	hepatocyte originated from H9	Human embryonic stem cell line
Adhesion	GEO	GSE78662	ENCFF336BBB	ENCFF695ZUU	A54	Cell line	G401	Kidney Rhabdoid tumor epithalial cell
Adhesion	GEO	GSE87996	ENCFF710WZN	ENCFF952OLL	A55	Cell line	HepG2	Hepatocellular carcinoma
Adhesion	GEO	GSE78626	ENCFF403NLY	ENCFF753GPU	A56	Primary Cell		Cardiac atrium fibroblast
Adhesion	GEO	GSE78628	ENCFF485LRB	ENCFF761PVR	A57	Cell line	NCI-H460	Lung carcinoma epithelial cell
Adhesion	GEO	GSE78660	ENCFF602JUN	ENCFF840QXH	A58	Primary Cell		Bronchus fibroblast of lung
Adhesion	GEO	GSE78658	ENCFF272LRD	ENCFF917KJE	A59	Cell line	Caki2	Kidney clear cell carcinoma epithelial cell
Adhesion	GEO	GSM1112818	ENCFF367QOQ	ENCFF958ZMB	A60	Invitro differentiated cell	ectodermal cell originated from HUES64	Human embryonic stem cell line with a normal 46XY karyotype
Adhesion	GEO	GSE78544	ENCFF001QZB	ENCFF001QZC	A61	Primary Cell		Renal cortical epithelial cell
Adhesion	GEO	GSE91305	ENCFF380VGD	ENCFF828WTU	A62	Cell line	A549	Lung carcinoma epithelial cell
Adhesion	GEO	GSE88362	ENCFF564BSM	ENCFF787PPA	A63	Cell line	HepG2	Hepatocellular carcinoma
Adhesion	GEO	GSM438363	ENCFF094HAH		A64	Cell line	IMR-90	Lung fibroblast
Adhesion	GEO	GSE88131	ENCFF090FUJ		A65	Invitro differentiated cell	smooth muscle cell originated from H9	Human embryonic stem cell line
Adhesion	GEO	GSE88131	ENCFF106HJE		A66	Invitro differentiated cell	smooth muscle cell originated from H9	Human embryonic stem cell line
Adhesion	GEO	GSE88167	ENCFF306XSY		A68	Invitro differentiated cell	cardiac muscle cell originated from RUES2	Human embryonic stem cell line with a normal 46XY karyotype
Adhesion	GEO	GSM438363	ENCFF381KYM		A69	Cell line	IMR-90	Lung fibroblast
Adhesion	GEO	GSE88601	ENCFF198SLH		A70	Invitro differentiated cell	hepatocyte originated from H9	Human embryonic stem cell line
Adhesion	GEO	GSM438363	ENCFF105OLM		A71	Cell line	IMR-90	Lung fibroblast
Adhesion	GEO	GSE78387	ENCFF002BHK		A72	Primary Cell	pyramidal neuron	Pyramidal neuron
Adhesion	GEO	GSE88601	ENCFF385OMY		A73	Invitro differentiated cell	hepatocyte originated from H9	Human embryonic stem cell line
Adhesion	GEO	GSM958739	ENCFF000DMW		A74	Cell line	HeLa-S3	Cervix adenocarcinoma epithelial cell
Adhesion	GEO	GSM1101681	ENCFF224MVX		A75	Primary Cell	fibroblast of skin of abdomen male embryo	Fibroblast of skin of abdomen
Adhesion	GEO	GSE93448	ENCFF000MRA		A76	Cell line	BE2C	Neuroblast of neuroblastoma
Adhesion	GEO	GSE93450	ENCFF000MTJ		A77	Cell line	Panc1	Pancreas epithelioid carcinoma epithelial cell
Adhesion	GEO	GSE88601	ENCFF795SKU		A78	Invitro differentiated cell	hepatocyte originated from H9	Human embryonic stem cell line
Adhesion	GEO	GSM438363	ENCFF547LGQ		A79	Cell line	IMR-90	Lung fibroblast
Adhesion	GEO	GSE90176	ENCFF000MVZ		A82	Cell line	U-87 MG	Brain glioblastoma-like epithelial cell
Adhesion	GEO	GSM1101666	ENCFF583FQV		A83	Primary Cell		Fibroblast of skin of back
Adhesion	GEO	GSE88167	ENCFF852LNM		A84	Invitro differentiated cell	cardiac muscle cell originated from RUES2	Human embryonic stem cell line with a normal 46XY karyotype
Adhesion	GEO	GSE93451	ENCFF000MTO		A85	Cell line	PFSK-1	Malignant primitive neuroectodermal tumor fibroblast
Adhesion	GEO	GSE88167	ENCFF301BEB		A86	Invitro differentiated cell	cardiac muscle cell originated from RUES2	Human embryonic stem cell line with a normal 46XY karyotype
Adhesion	GEO	GSE88167	ENCFF335BVH		A87	Invitro differentiated cell	cardiac muscle cell originated from RUES2	Human embryonic stem cell line with a normal 46XY karyotype
Adhesion	GEO	GSM438361	ENCFF476PHC		A88	Cell line	H1	Human embryonic stem cell line
Adhesion	GEO	GSM438361	ENCFF712SHP		A89	Cell line	H1	Human embryonic stem cell line
Adhesion	GEO	GSM438361	ENCFF793PLC		A90	Cell line	H1	Human embryonic stem cell line
Adhesion	GEO	GSM438361	ENCFF816QOY		A91	Cell line	H1	Human embryonic stem cell line
Adhesion	GEO	GSM438361	ENCFF821YWS		A92	Cell line	H1	Human embryonic stem cell line
Adhesion	GEO	GSE78672	ENCFF464PFE		A95	Primary Cell		Airway epithelial cell
Adhesion	GEO	GSE78672	ENCFF987MZF		A99	Primary Cell		Airway epithelial cell
Suspension	GEO	GSE90268	ENCFF000ESP	ENCFF000ESV	S1	Primary Cell		B cell
Suspension	GEO	GSE90268	ENCFF000ESQ	ENCFF000ESW	S2	Primary Cell		B cell
Suspension	GEO	GSE90268	ENCFF000ESR	ENCFF000ESY	S3	Primary Cell		B cell
Suspension	GEO	GSE78605	ENCFF000EUR	ENCFF000EUS	S4	Primary Cell		Hematopoeticel multipotent progenitor
Suspension	GEO	GSE90272	ENCFF000HUU	ENCFF000HVA	S5	Primary Cell		CD14-positive monocyte
Suspension	GEO	GSE90272	ENCFF000HUV	ENCFF000HVB	S6	Primary Cell		CD14-positive monocyte
Suspension	GEO	GSE90272	ENCFF000HUW	ENCFF000HVC	S7	Primary Cell		CD14-positive monocyte
Suspension	GEO	GSE78558	ENCFF001RCT	ENCFF001RDB	S8	Cell line	K562	Chronic myelogenous leukemia lymphoblast
Suspension	GEO	GSE78621	ENCFF002DLR	ENCFF002DLS	S9	Cell line	OCI-LY7	B cell lymphoma
Suspension	GEO	GSE78642	ENCFF002DLT	ENCFF002DLU	S10	Cell line	Karpas-422	B cell non-hodgkin lymphoma
Suspension	GEO	GSM1060237	ENCFF158OUQ	ENCFF214MGP	S11	Primary Cell		CD8-positive, alpha-beta T cell
Suspension	GEO	GSM979634	ENCFF000TUP		S14	Cell line	B cell	B cell
Suspension	GEO	GSM979635	ENCFF000TVA		S15	Primary Cell		Hematopoeticel multipotent progenitor cell
Suspension	GEO	GSE88583	ENCFF481BWJ		S17	Cell line	GM12878	B lymphocyte
Suspension	GEO	GSE88583	ENCFF974EKR		S18	Cell line	GM12878	B lymphocyte
Suspension	GEO	GSM1059487	ENCFF141XOX	ENCFF861MLB	S19	Primary Cell		CD4-positive, alpha-beta T cell
Suspension	GEO	GSM1060238	ENCFF249TVM	ENCFF570NFU	S21	Primary Cell		CD4-positive, alpha-beta T cell
Suspension	GEO	GSM1220575	ENCFF246VSY	ENCFF709TXQ	S22	Primary Cell		CD14-positive monocyte
Suspension	GEO	GSE88622	ENCFF444KCV	ENCFF606ZTR	S23	Cell line	K562	Chronic myelogenous leukemia lymphoblast
