# Supplementary material for: Reprogramming anchorage dependency by adherent-to-suspension transition promotes metastatic dissemination
Source: Mol Cancer. 2023 Mar 30;22:63. doi: 10.1186/s12943-023-01753-7 (PMC10061822; doi:10.1186/s12943-023-01753-7)
Supplement: Supplementary file 10 — Additional file 10: Table S2. Microarray data of adhesion and suspension cells used in this study from Human Protein Atlas (HPA). [file 12943_2023_1753_MOESM10_ESM.pdf]

Table S2. Microarray data of adhesion and suspension cells used in this study from Human Protein Atlas (HPA

		Analyzed_Samples		
Group	Data Source	labeling	Biosample Type	Cell line
Adhesion	Human Protein Atlas (HPA)	A91	Cell line	A-431
Adhesion	Human Protein Atlas (HPA)	A92	Cell line	A549
Adhesion	Human Protein Atlas (HPA)	A93	Cell line	AF22
Adhesion	Human Protein Atlas (HPA)	A94	Cell line	AN3-CA
Adhesion	Human Protein Atlas (HPA)	A95	Invitro differentiated cell	ASC diff
Adhesion	Human Protein Atlas (HPA)	A96	Invitro differentiated cell	ASC TERT1
Adhesion	Human Protein Atlas (HPA)	A97	Cell line	BEWO
Adhesion	Human Protein Atlas (HPA)	A98	Cell line	BJ
Adhesion	Human Protein Atlas (HPA)	A99	Invitro differentiated cell	BJ hTERT+
Adhesion	Human Protein Atlas (HPA)	A100	Invitro differentiated cell	BJ hTERT+ SV40 Large T+
Adhesion	Human Protein Atlas (HPA)	A101	Invitro differentiated cell	BJ hTERT+ SV40 Large T+ RasG12V
Adhesion	Human Protein Atlas (HPA)	A102	Cell line	CACO-2
Adhesion	Human Protein Atlas (HPA)	A103	Cell line	CAPAN-2
Adhesion	Human Protein Atlas (HPA)	A104	Cell line	EFO-21
Adhesion	Human Protein Atlas (HPA)	A105	Invitro differentiated cell	fHDF/TERT166
Adhesion	Human Protein Atlas (HPA)	A106	Cell line	GAMG
Adhesion	Human Protein Atlas (HPA)	A107	Cell line	HaCaT
Adhesion	Human Protein Atlas (HPA)	A108	Invitro differentiated cell	HAP1
Adhesion	Human Protein Atlas (HPA)	A109	Cell line	HBEC3-KT
Adhesion	Human Protein Atlas (HPA)	A110	Cell line	HBF TERT88
Adhesion	Human Protein Atlas (HPA)	A111	Cell line	HEK 293
Adhesion	Human Protein Atlas (HPA)	A112	Cell line	HeLa
Adhesion	Human Protein Atlas (HPA)	A113	Cell line	Hep G2
Adhesion	Human Protein Atlas (HPA)	A114	Cell line	HHSteC
Adhesion	Human Protein Atlas (HPA)	A115	Cell line	HMC-1
Adhesion	Human Protein Atlas (HPA)	A116	Cell line	HSkMC
Adhesion	Human Protein Atlas (HPA)	A117	Cell line	hTCEpi
Adhesion	Human Protein Atlas (HPA)	A118	Cell line	hTEC/SVTERT24-B
Adhesion	Human Protein Atlas (HPA)	A119	Cell line	hTERT-HME1
Adhesion	Human Protein Atlas (HPA)	A120	Cell line	hTERT-RPE1
Adhesion	Human Protein Atlas (HPA)	A121	Cell line	HUVEC TERT2
Adhesion	Human Protein Atlas (HPA)	A122	Cell line	LHCN-M2
Adhesion	Human Protein Atlas (HPA)	A123	Cell line	MCF7
Adhesion	Human Protein Atlas (HPA)	A124	Cell line	NTERA-2
Adhesion	Human Protein Atlas (HPA)	A125	Cell line	OE19
Adhesion	Human Protein Atlas (HPA)	A126	Cell line	PC-3
Adhesion	Human Protein Atlas (HPA)	A127	Cell line	RH-30
Adhesion	Human Protein Atlas (HPA)	A128	Cell line	RPTEC TERT1
Adhesion	Human Protein Atlas (HPA)	A129	Cell line	RT4
Adhesion	Human Protein Atlas (HPA)	A130	Cell line	SiHa
Adhesion	Human Protein Atlas (HPA)	A131	Cell line	SK-BR-3
Adhesion	Human Protein Atlas (HPA)	A132	Cell line	SK-MEL-30
Adhesion	Human Protein Atlas (HPA)	A133	Cell line	SuSa
Adhesion	Human Protein Atlas (HPA)	A134	Cell line	T-47d
Adhesion	Human Protein Atlas (HPA)	A135	Cell line	TIME
Adhesion	Human Protein Atlas (HPA)	A136	Cell line	U-138 MG
Adhesion	Human Protein Atlas (HPA)	A137	Cell line	U-2 OS
Adhesion	Human Protein Atlas (HPA)	A138	Cell line	U-2197
Adhesion	Human Protein Atlas (HPA)	A139	Cell line	U-251 MG
Adhesion	Human Protein Atlas (HPA)	A140	Cell line	U-87 MG
Adhesion	Human Protein Atlas (HPA)	A141	Cell line	WM-115
Suspension	Human Protein Atlas (HPA)	S22	Cell line	Daudi
Suspension	Human Protein Atlas (HPA)	S23	Cell line	HDLM-2
Suspension	Human Protein Atlas (HPA)	S24	Cell line	HEL
Suspension	Human Protein Atlas (HPA)	S25	Cell line	HL-60
Suspension	Human Protein Atlas (HPA)	S26	Cell line	JURKAT
Suspension	Human Protein Atlas (HPA)	S27	Cell line	K-562
Suspension	Human Protein Atlas (HPA)	S28	Cell line	Karpas-707
Suspension	Human Protein Atlas (HPA)	S29	Cell line	MOLT-4
Suspension	Human Protein Atlas (HPA)	S30	Cell line	NB-4
Suspension	Human Protein Atlas (HPA)	S31	Cell line	REH
Suspension	Human Protein Atlas (HPA)	S32	Cell line	RPMI-8226
Suspension	Human Protein Atlas (HPA)	S33	Cell line	SCLC-21H
Suspension	Human Protein Atlas (HPA)	S34	Cell line	SH-SY5Y
Suspension	Human Protein Atlas (HPA)	S35	Cell line	THP-1
Suspension	Human Protein Atlas (HPA)	S36	Cell line	U-266/70
Suspension	Human Protein Atlas (HPA)	S37	Cell line	U-266/84
Suspension	Human Protein Atlas (HPA)	S38	Cell line	U-698
Suspension	Human Protein Atlas (HPA)	S39	Cell line	U-937

A)

Origin
Lymphoblast
Epidermis of epidermoid carcinoma patient
Neuroepithelial-like stem cell
cervical cancer cell
Adipose stromal cell
Adipose stromal cell
Placenta of choriocarcinoma patient
Normal foreskin of neonatal
Normal foreskin of neonatal
Normal foreskin of neonatal
Normal foreskin of neonatal
Colon epithelial cell of colorectal adenocarcinoma patient
Pancreatic ductal adenocarcinoma
Ovary cystadenocarcinoma
Dermal fibroblast
Glioma cell
Epithelial keratinocyte
Leukemia cell
hTERT-immortalized cell
Brain fibroblast
Embryonic kidney cell
Cervix epithelial cell
Hepatocellular carcinoma
Liver
Mast cell leukemia
Skeletal muscle cell
Corneal epithelial cell
Thymic epithelial cell
Breast epithelial cell
Retinal pigment epithelial cell
Vascular endothelial cell
Skeletal myoblast cell
Breast adenocarcinoma
Teratocarcinoma cell
Adenocarcinoma
Prostate adenocarcinoma
Alveolar rhabdomyosarcoma
Renal proximal tubule epithelial cell
Bladder transitional cell papilloma
Squamous cell carcinoma
Adenocarcinoma
Melanoma
Testicuar germ cell
Breast carcinoma epithelial cell
Neonatal foreskin microvascular endothelial cell
Brain glioblastoma
Osteosarcoma
Fibrous histiocytma
Glioblastoma
Glioma cell
Melanoma
Burkitt's lymphoma
Hodgkin lymphoma
Erythroleukemia
Promyelocytic leukemia
T lymphocyte cell
Myelogenous leukemia
Myeloma
Lymphoblast
Leukemia cell
Lymphocytic leukemia
B lymphocyte
Lung carcinoma
Neuroblastoma
Monocyte
Myeloma
Myeloma
B lymphocyte
Myeloid leukemia cell
