# Supplementary material for: Reprogramming anchorage dependency by adherent-to-suspension transition promotes metastatic dissemination
Source: Mol Cancer. 2023 Mar 30;22:63. doi: 10.1186/s12943-023-01753-7 (PMC10061822; doi:10.1186/s12943-023-01753-7)
Supplement: Supplementary file 11 — Additional file 11: Table S3. 100 transcription factors upregulated in suspension cells compared to adhesion cells. [file 12943_2023_1753_MOESM11_ESM.pdf]

Table S. 100 transcription factors upregulated in suspension cells compared to adhesion cells

LOCUS	Difference	Fold_Change	P.VAL	FDR	Bonferroni
IKZF1	6.491829256	89.28670455	0	0	0
IKZF3	4.052722342	49.39507689	0	0	0
SPI1	6.071040526	32.95260822	0	0	0
POU2AF1	3.214411448	27.93988207	6.44E-15	3.7202E-10	4.4987E-13
CEBPE	1.713355165	21.61633382	0	0	0
GFI1B	1.604525691	19.48737395	1.40E-09	8.087E-05	3.7267E-08
SCML4	1.964896512	16.83116971	1.23E-11	7.1021E-07	4.9252E-10
PAX5	2.995897038	15.80942479	1.01E-12	5.8227E-08	4.9513E-11
HSF5	1.234096774	15.02532438	7.73E-14	4.4642E-09	4.5322E-12
IRF8	5.211902533	12.0562409	0	0	0
IRF4	3.670005228	10.69497608	1.33E-15	7.6969E-11	1.0276E-13
TBX21	2.044405953	10.52643299	7.72E-12	4.4598E-07	3.2482E-10
BATF	4.626811705	9.861355258	0	0	0
ZNF831	1.490462928	9.060512315	7.28E-11	4.2056E-06	2.5063E-09
KLF1	1.509797155	9.014868978	1.61E-07	0.00930859	2.7714E-06
SPIB	3.418673975	8.898964825	0	0	0
SP140	2.954092325	6.713033649	0	0	0
ZNF80	0.911970989	6.496365883	1.13E-09	6.5384E-05	3.0539E-08
TFEC	2.264995093	5.22899186	1.60E-09	9.2646E-05	4.2131E-08
RUNX3	4.452805483	5.131106847	0	0	0
NFE2	3.009628259	4.782028339	5.85E-10	3.3779E-05	1.6714E-08
MYB	3.016963369	4.692885937	4.69E-14	2.7067E-09	2.8436E-12
POU2F2	3.767806198	4.344674984	0	0	0
GFI1	1.996058396	4.190861526	3.41E-10	1.9672E-05	1.0214E-08
ESR2	1.125673771	4.15471402	1.11E-08	0.00064128	2.4458E-07
ASCL2	1.728545476	4.096141624	3.24E-08	0.0018703	6.4599E-07
LYL1	4.322417017	3.640794611	6.66E-16	3.8485E-11	5.3525E-14
IRF5	3.436626509	3.626027789	1.28E-12	7.3839E-08	6.1894E-11
FLI1	3.821756827	3.142319597	1.09E-12	6.3243E-08	5.3596E-11
EAF2	3.730425439	3.071457392	0	0	0
ZMAT1	2.093751603	3.020334542	3.59E-11	2.075E-06	1.3174E-09
ZBTB32	1.424701698	2.817381016	5.35E-08	0.00309151	1.0227E-06
TFAP2E	1.352879358	2.637903485	1.28E-09	7.3725E-05	3.4148E-08
ZNF296	2.136402212	2.515852305	1.68E-09	9.7275E-05	4.4116E-08
NFATC2	2.374304591	2.496814166	6.45E-09	0.00037286	1.501E-07
SP110	3.034830748	2.46024676	0	0	0
MEF2B	1.826625322	2.446937	4.87E-08	0.00281249	9.3781E-07
HHEX	2.89755765	2.444196306	8.48E-10	4.902E-05	2.3578E-08
STAT5A	3.596704314	2.303312387	0	0	0
KLF2	3.645210307	2.235962456	7.03E-09	0.00040612	1.6238E-07
AKNA	3.447066711	2.218151742	0	0	0
NFATC1	2.193823296	2.100616745	1.72E-10	9.9449E-06	5.4975E-09
TBX19	1.237123635	2.086190348	8.76E-10	5.06E-05	2.428E-08
TCF7	2.009163881	2.02335196	4.46E-08	0.00257689	8.6502E-07
ZNF736	1.943739356	2.007519196	1.78E-08	0.00103075	3.7509E-07
CCDC17	1.050493318	1.99169818	2.50E-08	0.00144472	5.105E-07
SNAI3	1.872805821	1.956158218	3.19E-11	1.8427E-06	1.1843E-09
ZNF169	1.49777445	1.950942641	0	0	0
IRF1	2.85828495	1.950354197	2.89E-15	1.6677E-10	2.111E-13

TFEB	2.019268598	1.859725489	1.09E-08	0.00063096	2.4119E-07
REL	2.297404564	1.857258728	0	0	0
ZNF345	1.257552636	1.8340775	5.75E-09	0.00033238	1.349E-07
NRL	0.902779207	1.80123437	1.87E-07	0.01079889	3.165E-06
SP140L	2.08489528	1.791727968	1.81E-09	0.00010448	4.7128E-08
ZNF341	1.373672574	1.751415909	7.07E-11	4.0862E-06	2.4439E-09
ZNF600	1.530831978	1.729413288	1.71E-07	0.00987335	2.9211E-06
ZNF107	1.712633334	1.716116082	9.66E-08	0.00557871	1.7494E-06
IRF7	2.463913246	1.710520864	1.15E-09	6.6686E-05	3.1107E-08
ARID5A	2.125424304	1.690451513	7.64E-11	4.4154E-06	2.6236E-09
BTG2	3.242472265	1.653935837	3.82E-10	2.2074E-05	1.1367E-08
ZNF589	1.992423728	1.646789243	1.29E-11	7.4692E-07	5.1619E-10
ZNF100	1.395805706	1.611366623	1.06E-07	0.00610289	1.8971E-06
ZNF789	1.407584597	1.597077669	2.31E-12	1.3317E-07	1.0645E-10
ZNF276	1.776961545	1.521039181	1.10E-11	6.3771E-07	4.4689E-10
ZNF430	1.294606441	1.489282153	2.29E-10	1.3209E-05	7.0787E-09
IKZF5	1.508223531	1.474471364	2.13E-12	1.228E-07	9.9196E-11
ZBTB25	1.185805551	1.45662465	2.86E-10	1.6496E-05	8.6775E-09
ZNF266	1.687229736	1.455215608	0	0	0
SETDB2	1.353282376	1.448231743	1.25E-09	7.2309E-05	3.3508E-08
ELF1	2.073260288	1.43078095	7.26E-12	4.1943E-07	3.066E-10
ZBTB48	1.435215843	1.398196237	5.59E-13	3.2301E-08	2.8484E-11
ZBTB49	1.031442802	1.396379486	1.79E-11	1.036E-06	6.9335E-10
ZNF706	1.525313004	1.394963761	3.74E-09	0.00021595	9.0506E-08
ZNF431	1.209745376	1.390683879	1.03E-07	0.00594826	1.8538E-06
ZNF76	1.347973674	1.378316093	1.05E-09	6.0631E-05	2.8439E-08
ZNF586	1.267647608	1.373988589	2.40E-09	0.00013847	6.0655E-08
NFATC3	1.550038023	1.369007045	2.96E-13	1.7126E-08	1.5784E-11
ZNF559	1.050998736	1.368791296	5.59E-08	0.00322919	1.0626E-06
ZNF26	1.048043993	1.367876982	1.07E-08	0.00062084	2.376E-07
ZNF641	1.198208329	1.366748001	1.45E-08	0.00083873	3.1122E-07
IRF2	1.599440185	1.355149656	5.57E-11	3.2195E-06	1.9715E-09
PHF21A	1.160177862	1.354213862	8.16E-08	0.00471586	1.5E-06
STAT5B	1.770270746	1.352650557	3.82E-12	2.2068E-07	1.7028E-10
ZNF783	1.122195944	1.346744509	3.31E-09	0.00019133	8.1278E-08
ZNF335	1.286985219	1.338148657	4.25E-08	0.00245763	8.2832E-07
STAT6	1.830258871	1.33543042	7.32E-08	0.00422713	1.3557E-06
ATF6B	1.599003463	1.320529417	1.75E-10	1.0113E-05	5.5812E-09
ZNF800	1.18102051	1.305875105	1.02E-07	0.00589342	1.8394E-06
HINFP	0.885850432	1.302111673	1.50E-07	0.00865904	2.5933E-06
ZNF394	1.29210175	1.298693651	1.64E-13	9.4672E-09	9.1294E-12
ZBTB40	1.162136986	1.274385528	4.39E-11	2.5382E-06	1.5913E-09
NFYC	1.134236286	1.26155477	5.45E-09	0.00031478	1.2827E-07
MAX	1.228137086	1.243966071	2.22E-16	1.2828E-11	1.9291E-14
IRF3	1.249569187	1.243209539	1.04E-09	6.0292E-05	2.832E-08
RFX5	1.292676157	1.239019912	1.00E-10	5.796E-06	3.3542E-09
NRF1	0.908574734	1.228866541	1.77E-08	0.00102199	3.7231E-07
MBD4	1.104039833	1.216077782	9.16E-11	5.2903E-06	3.0883E-09
GTF3A	1.095010959	1.168241488	1.17E-10	6.7705E-06	3.8556E-09
MLX	0.753018068	1.146867878	3.84E-08	0.00222008	7.559E-07
ZNF317	0.647877436	1.139606082	1.26E-07	0.00727741	2.2201E-06
