# Supplementary material for: Reprogramming anchorage dependency by adherent-to-suspension transition promotes metastatic dissemination
Source: Mol Cancer. 2023 Mar 30;22:63. doi: 10.1186/s12943-023-01753-7 (PMC10061822; doi:10.1186/s12943-023-01753-7)
Supplement: Supplementary file 6 — Additional file 6: Figure S5. Induction of AST factors in human and mouse model-derived CTCs and LVI. [file 12943_2023_1753_MOESM6_ESM.pdf]

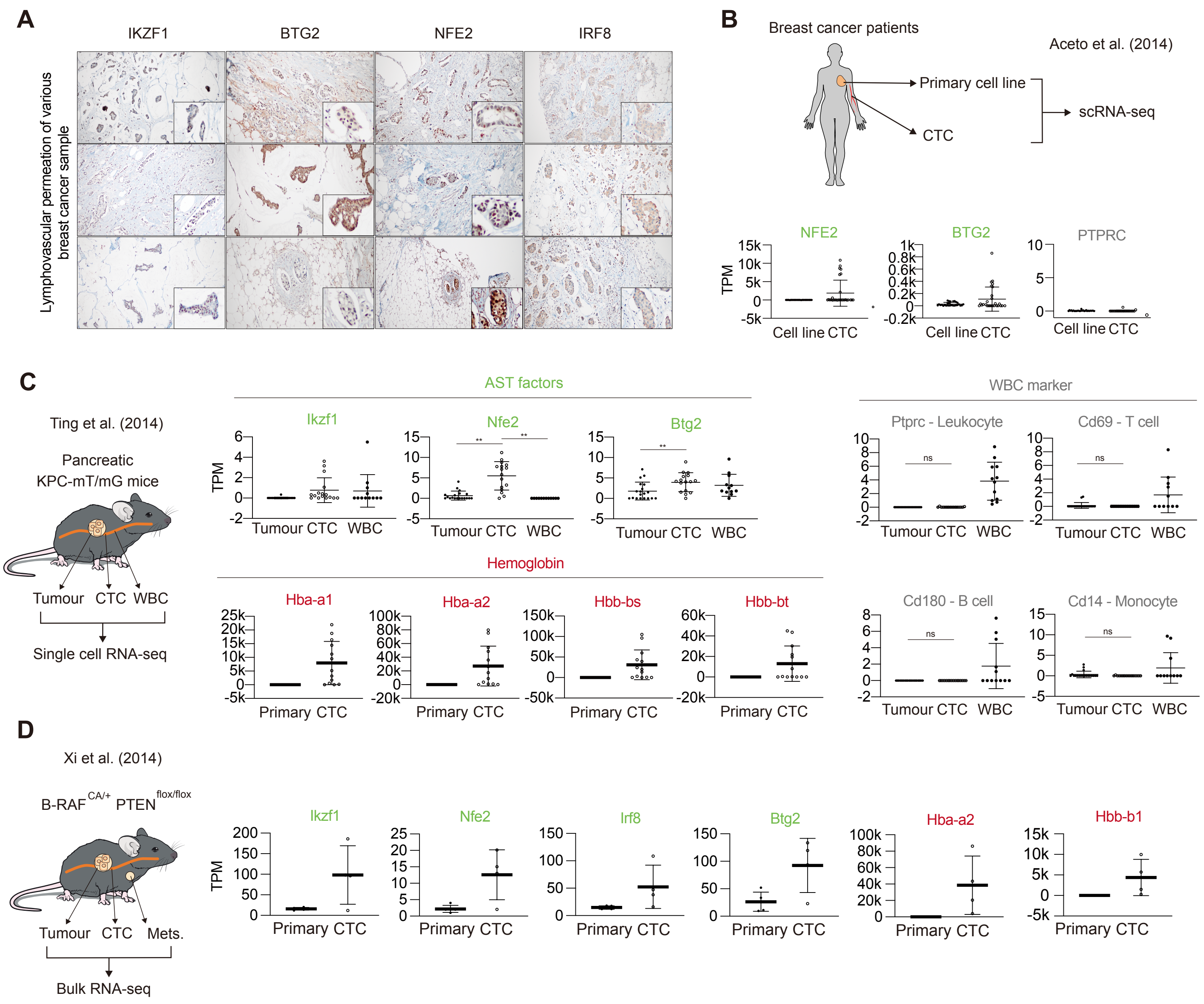
A
IKZF1
BTG2
NFE2
IRF8
Lymphovascular permeation of various breast cancer sample
B
Breast cancer patients
Aceto et al. (2014)
Primary cell line
CTC
scRNA-seq
NFE2
BTG2
PTPRC
TPM
Cell line CTC
C
Ting et al. (2014)
Pancreatic
KPC-mT/mG mice
Tumour CTC WBC
Single cell RNA-seq
AST factors
Ikzf1
Nfe2
Btg2
WBC marker
Ptprc - Leukocyte
Cd69 - T cell
Hemoglobin
Hba-a1
Hba-a2
Hbb-bs
Hbb-bt
Cd180 - B cell
Cd14 - Monocyte
Tumour CTC WBC
Primary CTC
ns
D
Xi et al. (2014)
B-RAF CA/+ PTEN flox/flox
Tumour CTC Mets.
Bulk RNA-seq
Ikzf1
Nfe2
Irf8
Btg2
Hba-a2
Hbb-b1
Primary CTC
